# Amphiphilic Polypeptides Obtained by Post-Polymerization Modification of Poly-l-Lysine as Systems for Combined Delivery of Paclitaxel and siRNA

**DOI:** 10.3390/pharmaceutics15041308

**Published:** 2023-04-21

**Authors:** Apollinariia Dzhuzha, Erik Gandalipov, Viktor Korzhikov-Vlakh, Elena Katernyuk, Natalia Zakharova, Sergey Silonov, Tatiana Tennikova, Evgenia Korzhikova-Vlakh

**Affiliations:** 1 Institute of Chemistry, Saint-Petersburg State University, Universitetsky Pr. 26, St. Petersburg 198504, Russia; 2 Institute of Macromolecular Compounds, Russian Academy of Sciences, Bolshoy Pr. 31, St. Petersburg 199004, Russia; 3 International Institute of Solution Chemistry and Advanced Materials Technologies, ITMO University, Lomonosov Street 9, St. Petersburg 191002, Russia; 4 Institute of Cytology, Russian Academy of Sciences, Tihkorezky Pr. 4, St. Petersburg 194064, Russia

**Keywords:** amphiphilic copolymers, polypeptides, polymer particles, drug delivery systems, dual-drug delivery, paclitaxel, siRNA

## Abstract

The development of effective anti-cancer therapeutics remains one of the current pharmaceutical challenges. The joint delivery of chemotherapeutic agents and biopharmaceuticals is a cutting-edge approach to creating therapeutic agents of enhanced efficacy. In this study, amphiphilic polypeptide delivery systems capable of loading both hydrophobic drug and small interfering RNA (siRNA) were developed. The synthesis of amphiphilic polypeptides included two steps: (i) synthesis of poly-αl-lysine by ring-opening polymerization and (ii) its post-polymerization modification with hydrophobic l-amino acid and l-arginine/l-histidine. The obtained polymers were used for the preparation of single and dual delivery systems of PTX and short double-stranded nucleic acid. The obtained double component systems were quite compact and had a hydrodynamic diameter in the range of 90–200 nm depending on the polypeptide. The release of PTX from the formulations was studied, and the release profiles were approximated using a number of mathematical dissolution models to establish the most probable release mechanism. A determination of the cytotoxicity in normal (HEK 293T) and cancer (HeLa and A549) cells revealed the higher toxicity of the polypeptide particles to cancer cells. The separate evaluation of the biological activity of PTX and anti-GFP siRNA formulations testified the inhibitory efficiency of PTX formulations based on all polypeptides (IC_50_ 4.5–6.2 ng/mL), while gene silencing was effective only for the Tyr-Arg-containing polypeptide (56–70% GFP knockdown).

## 1. Introduction

Currently, the joint delivery of anticancer drugs has received a lot of attention due to the increased anti-tumor efficacy compared to the administration of single-drug systems [1,2]. To date, there have been a number of publications on the development of dual-drug delivery systems of doxorubicin and paclitaxel based on graphene nanoparticles and graphene oxide [3], 177-Lu-bombesin and paclitaxel delivery systems [4] and afatinib and paclitaxel based on poly(lactide-*co*-glycolide) (PLGA) [5], paclitaxel and cisplatin delivery systems based on micellar systems derived from poly(2-oxazoline) [6], or nanotubes based on triple amphiphilic block copolymer poly(ethylene glycol)-*b*-polylactide-*b*-poly(ethylene glycol) [7].

In addition to the combination of traditional chemotherapeutic agents with each other, there are also ongoing investigations in the field of complex therapy, combining chemotherapeutic substances with protein or gene-therapy agents [8,9,10]. Possible synergistic effects caused by actions on several targets in tumor cells and tissues can lead to an increased efficiency of tumor inhibition, a decrease in the dose of toxic drugs and side effects, and, as a consequence, improved survival. The use of cytokines and antibodies, as well as small interfering RNA (siRNA) in cancer therapy, is a relatively new and rapidly developing area [11,12]. The action of proteins is connected either with the direct induction of apoptosis in tumor cells through specific pathways or is caused by indirect effects of tumor inhibition by immune response stimulation or tumor targeting [13,14]. Currently, there are a number of monoclonal antibodies approved by the Food and Drug Administration (FDA), as well as undergoing clinical trials for the treatment of various cancers [15]. It has been shown that the combination of antibodies with chemotherapeutic drugs leads to significant synergistic effects in cancer therapy [16]. For example, trastuzumab is known to inhibit the proliferation of HER2-overexpressing human cancer tumors. Combined treatment regimens of trastuzumab and conventional chemotherapeutic agents such as paclitaxel, doxorubicin, cisplatin, and cyclophosphamide lead to an increased antitumor efficacy [17].

A significant problem in the therapy of solid tumors is their ability to evade the immune system. One of the reasons for this is the vascular abnormalities caused by increased levels of proangiogenic factors such as vascular epithelial growth factor (VEGF) and angiopoietin 2 (ANG2). The drug bevacizumab (Avastin^®^), which specifically binds to VEGF and suppresses its activity, has been approved for clinical use as an angiogenesis inhibitor [18], resulting in decreased vascularization and tumor apoptosis [19]. However, many patients become resistant to treatment, and no clinical or biological factors have been identified that can clearly predict which patients will react well to bevacizumab and which will be resistant to it.

Besides immunotherapy, special attention is paid to the development of gene therapeutic substances. In particular, small interfering RNAs (siRNAs) and microRNAs (miRNAs), which can suppress the posttranslational stage of gene expression, are of significant interest [18]. In this case, there is no interaction with DNA—which avoids mutations and reduces teratogenic risks. Moreover, si/miRNAs act in small amounts and are characterized by high efficiency. One of the main obstacles limiting the use of si/miRNAs is their instability under physiological conditions due to their fast destruction by serum nucleases. Furthermore, the negative charge of nucleic acids limits their entrance into cells. Therefore, a therapy with gene-therapeutic agents requires the use of delivery systems.

A number of studies have shown that the simultaneous delivery of anti-cancer drugs and siRNA through the same delivery system appears to be more effective than sequentially administering two separate single-drug formulations [20,21,22]. In recent years, delivery systems that allow simultaneous delivery of the chemotherapeutic drug and siRNA to the tumor are considered to be one of the most promising tools in cancer treatment [23,24]. However, the development of delivery systems combining together such chemically different substances as small cytostatic drugs (often uncharged or hydrophobic) and negatively charged siRNA poses a great challenge. It is known that cationic liposomes, polymers (polyethyleneimine (PEI), polylysine, polyarginine, chitosan and its derivatives), and dendrimers (polyamidoamine, abbreviated as PAMAM) are the best non-viral candidates for the delivery of nucleic acids [25,26,27]. These carriers form interpolyelectrolyte complexes with nucleic acids, resulting in increased intracellular transport and stability to cleavage by nucleases [28]. However, they are poorly suited to the encapsulation of many common anticancer drugs. In order to overcome this drawback, various solutions have been proposed to develop combined delivery systems that effectively encapsulate cytostatic drugs and bind siRNA.

In particular, the composition of PLGA- and PEI-protected gold nanoparticles was investigated by Kumar et al. for codelivery of doxorubicin and EGFP siRNA [1]. Alinejad et al. reported the development of a formulation based on chitosan-carboxymethyl dextran nanoparticles for codelivery of doxorubicin and IL17RB siRNA [29]. In experiments on the MDA-MB361 cell line (human breast cancer cells), it was shown that two-component systems were superior to single-component and free doxorubicin. Biswas et al. proposed an siRNA and doxorubicin delivery system based on cationic dendrimers modified by PEG and lipids [30]. The efficacy of siRNA delivery was demonstrated by suppression of GFP (green fluorescent protein) expression in C166-GFP cells, which was assessed by the disappearance of GFP fluorescence. Zhu et al. reported the creation of cationic micelles based on conjugates of cationic dendrimers and polycaprolactone containing paclitaxel and GFP siRNA [31]. The efficacy of intracellular delivery was evaluated by the suppression of GFP expression in GFP-expressing MDA-MB-435 cells. Chen et al. developed a VEGF siRNA and doxorubicin delivery system based on calcium phosphate nanoparticles encapsulated into cationic liposomes [32]. In this case, doxorubicin was loaded into calcium phosphate cores during nanoprecipitation, while siRNAs were bound to the cationic liposomes through ionic interactions. Evaluation of the obtained systems in vivo also revealed the best suppression of tumor growth when using two-component delivery systems.

Amphiphilic cationic copolymers represent one the most promising candidates for the joint delivery of hydrophobic chemotherapeutics and siRNA, since their hydrophobic and cationic segments are involved in the retention of the hydrophobic drug and nucleic acid, respectively [22,33]. Amphiphilic copolymers that are now widely utilized usually consist of hydrophobic aliphatic polyesters such as poly(ε-caprolactone) (PCL), PLGA and polylactide (PLA), and hydrophilic cationic polymers such as PEI, polylysine, chitosan, or their PEGylated derivatives [31,33,34,35]. Such amphiphilic copolymers form nanoparticles with siRNA that are more stable than simple polyplexes formed from polycations or their PEGylated forms with siRNA [36].

One of the promising classes of synthetic polymers for drug delivery is poly(amino acids) (or polypeptides) [37,38]. To date, various cationic polypeptides have been examined for the delivery of nucleic acids [39,40,41]. Recently, it was shown that cationic amphiphilic polypeptides can be successfully used for the delivery of nucleic acids [42,43,44,45,46]. At the same time, amphiphilic polypeptides can successfully serve as a delivery systems for cytostatic drugs [38,47,48,49]. Compared to other cationic polymers used for the delivery of nucleic acids, poly(amino acids) such as polylysine, polyarginine, and polyornithine are fully biodegradable into non-toxic and natural metabolites. In contrast to polyarginine, the side-chain primary amino groups of polylysine and polyornithine can be easily modified with different functional targets such as hydrophobic moieties, sugars, etc., to provide the desired design and properties of the resulting delivery system.

In this study, amphiphilic polypeptides obtained by post-polymerization modification of poly-l-lysine with hydrophobic amino acids and l-arginine/l-histidine were proposed for the combined loading of the hydrophobic cytostatic drug paclitaxel and siRNA. The synthesized copolymers were characterized with respect to their composition and molecular weight. The nanoparticles formed from the amphiphilic polypeptides were loaded with PTX and/or double-stranded nucleic acid and thoroughly characterized with regards to their physicochemical and biological properties.

## 2. Materials and Methods

### 2.1. Reagents, Biologicals, and Supplements

ε-Z-l-lysine, Fmoc-l-phenylalanine, Fmoc-l-valine, Fmoc-l-isoleucine, Fmoc-*O*-*tert*-butyl-l-tyrosine, *N*_α_-Fmoc-*N*′-Boc-l-tryptophan, *N*_α_-Fmoc-*N*′-trityl-l-histidine, N_α_-Fmoc-N_ω_-(4-methoxy-2,3,6-trimethylbenzenesulfonyl)-l-arginine, and other chemicals used for synthesis of the monomer and polypeptides, as well as for the deprotection of polypeptides, were purchased from Sigma–Aldrich (Darmstadt, Germany) and used as received. All organic solvents used for synthesis and other manipulations were received from Vecton Ltd. (St. Petersburg, Russia) and distilled prior to use.

Membrane filters (0.22 µm) produced by Millipore Sigma (St. Luis, MO, USA) were utilized for the ultrafiltration of all buffer solutions. The salts used for the preparation of buffer solutions were of analytical purity grade. Spectra/Pore^®^ dialysis bags (MWCO: 1000, Rancho Dominguez, CA, USA) and Orange Scientific dialysis bags (MWCO: 2000) (Braine-l’Alleud, Belgium) were applied for the purification of the polymers obtained. Amicon Ultra filter tubes with 3000 MWCO (0.5 mL) were purchased from Merck (Darmstadt, Germany).

Model non-labeled and TAMRA-labeled 23-base oligothymidine and oligoadenine were purchased from Biobeagle^TM^ (St. Petersburg, Russia). GFP-siRNA-sense and GFP-siRNA-antisense were obtained from DNA-Synthesis (Moscow, Russia). The siRNA sequence was the following: sense: 5′-GCAAGCUGACCCUGAAGUUdTdT-3′, antisense: 5′-AACUUCAGGGUCAGCUUGCdTdT-3′. GenJect™-39 and GenJect™-40 transfection agents were produced by MOLECTA (Moscow, Russia). Heparin sodium salt from porcine intestinal mucosa was a product of Sigma–Aldrich (Darmstadt, Germany). Agarose for gel electrophoresis was obtained from Rosmedbio Ltd. (St. Petersburg, Russia). Dulbecco’s modified Eagle’s medium (DMEM), RPMI-1640 cell culture medium, gentamicin, and fetal bovine serum (FBS) were obtained from BioloT (St. Petersburg, Russia).

The human embryonic kidney cell line (HEK 293T), human cervical carcinoma cells (HeLa), and human lung carcinoma cells (A549) were obtained from the cell line collection of the Institute of Cytology of Russian Academy of Sciences (St. Petersburg, Russia), while the K562 modified with d2EGFP plasmid cell line (K562/GFP) was gifted from Blokhin Cancer Research Center (Moscow, Russia).

All other materials are described further upon their appearance in the text.

### 2.2. Methods

#### 2.2.1. Synthesis of Copolymers

Ring-opening polymerization (ROP) of α-amino acid N-carboxyanhydrides was carried out to obtain poly(Z-lysine) according to the previously published procedure [50]. Briefly, Lyz(Z)-NCA monomer was prepared via the reaction of α-l-Lys(Z) with triphosgene under an argon atmosphere in anhydrous dioxane with the addition of α-pinene. Synthesized Lyz(Z) NCA was purified by recrystallization from anhydrous petroleum ether. Poly(Z-l-lysine)) (P[K(Z)] was synthesized by ROP with the use of a 4 wt% monomer solution in freshly distilled and anhydrous 1,4-dioxane at a monomer/*n*-hexylamine ratio equal to 150. Polymerization was carried out at 30 °C for 72 h in an argon atmosphere. The resulting polymer was precipitated by diethyl ether, washed several times with the same solvent, and then air-dried. The polypeptide yield was 93%. Molecular-weight characteristics were determined by the static light scattering method and also calculated from the ^1^H NMR spectrum of the synthetized polymer (P[K(Z)]).

In order to remove the carboxybenzyl (Z) protection of the ε-amino group of lysine, 200 mg of the polymer was dissolved in 4 mL of trifluoroacetic acid in an ice bath under stirring. After 30 min, 2 mL of a 33 wt% solution of hydrogen bromide in acetic acid was added to the reaction solution, left for 20 min under cooling, and then incubated for 3 h at room temperature. Afterwards, the reaction was stopped by polymer precipitation in cooled diethyl ether taken in a five-fold excess. The precipitate separated by centrifugation was dissolved in 10 mL of DMF. The solution of polymer was transferred to a dialysis bag (MWCO 1000) and left for dialysis for 48 h with sequential replacement of the medium: DMF/deionized water mixture (50/50, *v*/*v*), deionized water, 1 M sodium chloride solution, and deionized water. Finally, the polypeptide products were dried by lyophilization.

The modification of P[K] was carried out in two steps: modification with (i) hydrophobic amino acids and (ii) basic amino acids. In the first step, the preliminary activation of the carboxylic groups of Fmoc-derivatives of Phe/Val/Ile/Tyr(tBu)/Trp(Boc) was carried out using DIC and NHS in DMF. Fmoc-derivatives were taken as 30 mol% of the molar amount of lysine in the copolymer. The desired amount of the amino-acid derivative of interest was dissolved in DMF. Then, a two-fold molar excess of NHS over the amount of carboxyl groups was dissolved in DMF and added to the amino acid solution under moderate stirring. After 15 min, a DMF solution containing a 1.1-fold molar excess of DIC over the amount of carboxyl groups was added to the reaction mixture and the resulting solution was left in the cold for 40 min. Then, the activated amino-acid derivative was added to the 15 wt% solution of P[K] in DMF. The reaction of modification was left under stirring for 4 h at 22 °C. The copolymers were purified by dialysis against a DMF/H_2_O mixture (50/50, *v*/*v*) and then H_2_O for 36 h. The final polypeptide products were dried by lyophilization. The yields of the resulting polypeptides were 80–95%. The modification degree was evaluated by the ^1^H NMR spectra of obtained copolymers.

During the second step, the obtained copolymers were modified with L-arginine or L-histidine using Fmoc-Arg(Mtr)-OH and Fmoc-His(Trt)-OH derivatives via the same protocol. Amino acids were taken as 45 mol% (arginine) and 60 mol% (histidine) of the molar amount of lysine in the copolymer. The yields of the modified copolymers were in the range of 45–78%.

Finally, the Fmoc, Boc, Trt, and Mtr protective groups were removed. In particular, Fmoc-protective groups were removed by 20% *tert*-butylamine solution in DMF for 16 h at 22 °C. The Fmoc-deprotected polypeptides were precipitated with precooled diethyl ether taken in a five-fold excess. In order to remove the Boc- or Trt-protective group, copolymers were placed in an ice bath, dissolved in TFA, and left for 1.5 h. Cleavage of the Mtr protective group of Arg-containing copolymers was performed by a 9% TFMSA/TFA solution for 30 min under cooling in an ice bath and then for 2 h at room temperature. The deprotected products were precipitated with a five-fold excess of precooled diethyl ether, purified by dialysis from DMF to H_2_O, and freeze dried.

#### 2.2.2. Characterization of Copolymers

The removal of the protective groups was monitored by the disappearance of characteristic signals in the ^1^H NMR spectrum (Figure S1 of Supplementary Materials). The contents of hydrophobic amino acids in the copolymers were calculated from ^1^H NMR spectra (DMSO-d6, 25 °C) (Figure S2 of Supplementary Materials).

The molecular weights of macromolecules were measured by the static light scattering method in solutions in DMSO at 21 °C as described earlier [42]. The hydrodynamic radii (R_h-D_) of scattering macromolecules were calculated from the Stokes–Einstein equation. The *M_w_* of copolymers was determined by the Debye method.

The composition of the resulting polypeptides was determined by the quantitative HPLC analysis of free amino acids derived as a result of the total acidic hydrolysis of polypeptides. For the preparation of hydrolysates, 4 mL of a 6 M HCl solution containing 0.0001% phenol was added to the ampoule containing 2 mg of a polypeptide. The ampoule was purged with argon, sealed, and incubated at a temperature of 110 °C for 96 h. After hydrolysis, the solvent was evaporated several times by water to eliminate HCl and to reach a neutral pH. The final residue was diluted with 0.5 mL of water, transferred to a glass, and dried by lyophilization. The amino-acid quantitative HPLC analysis was performed by a previously developed protocol [51] at the Chemical Analysis and Materials Research Center of SPbU Research Park.

#### 2.2.3. Preparation and Characterization of Particles

Polymer particles were obtained using the nanoprecipitation method. For this purpose, 2 mg of polypeptides were preliminary dissolved in 100 μL of DMSO. Then, the obtained solution was added dropwise to deionized water (900 µL) under vigorous stirring (1000 rpm). The resulting dispersion was short-time ultrasonicated using a Sonopuls ultrasound probe (30%, 30 s) (Bandelin, Berlin, Germany) and left for 30 min for stabilization.

The particle characteristics (hydrodynamic diameter, *D_H_;* polydispersity index, PDI, and ζ-potential) were measured by dynamic and electrophoretic light scattering (DLS/ELS) at a scattering angle of 173° and 25 °C using a ZetasizerNano-ZS (Malvern, UK) equipped with an He–Ne laser at 633 nm.

The morphology of empty and drug-loaded polypeptide particles was evaluated by transmission electron microscopy (TEM). A Jeol JEM-2100 transmission electron microscope (Kyoto, Japan) and 300 mech Cu grids covered with carbon and thin formvar film were used for sample analysis. In order to prepare a sample for microscopy, an aqueous dispersion of polymer particles (1 mg/mL) was dropped at the surface of the grids, treated with a 2% (*w*/*v*) uranyl acetate solution for 30–60 s, and left in air for 24 h at 22 °C. ImageJ open software (the National Institute of Mental Health, Bethesda, MD, USA) was used to calculate the average particles’ diameter from the TEM images.

#### 2.2.4. Preparation and Characterization of PTX-Loaded Delivery Systems

PTX-loaded particles were obtained using the strategy described above. Firstly, the PTX solution in DMSO (1 mg/mL) was added to the polymer dissolved in DMSO (2 mg/mL), mixed well, and then the obtained solution was dropped into deionized water under intensive stirring (1000 rpm). The quantities of PTX and polymers solutions to be mixed were calculated to achieve 25, 50, 100, and 150 µg of PTX per 1 mg of polymer. After short ultrasonication treatment, the obtained dispersions were left in the cold for 30 min, and the characteristics of all the drug-loaded systems were examined by DLS.

In order to determine the amount of unloaded paclitaxel, free paclitaxel was removed via ultrafiltration with the use of ultrafiltration tubes with a molecular weight cutoff membrane of 3000 (Amicon Ultra, Sigma-Aldrich, Darmstadt, Germany) using washing with 10% acetonitrile solution. The filtrates were collected and freeze dried. The lyophilized samples were dissolved in acetonitrile and analyzed by quantitative reversed-phase HPLC using a previously reported protocol [51]. The analysis was carried out at the Chemical Analysis and Materials Research Centre of SPbU Research Park. In this case, the loading was calculated as the difference between the initial PTX amount and that determined in the washing solutions. In addition, the samples after ultrafiltration were transferred to acetonitrile and filtered. The filtrate was analyzed by the same method. The results were coincided for indirect and direct methods of the PTX-loading determination.

#### 2.2.5. Preparation and Characterization of Oligo-dT-dA-Loaded Delivery Systems

Due to the positive surface charge of the obtained polypeptides, they are capable of binding negative oligonucleotides. An oligo-thymidine and oligo-adenine duplex of 23-base pairs (oligo-dT-dA) was used as a stable physicochemical model of siRNA. In order to obtain the oligo-dT-dA duplex, oligo-dT and oligo-dA solutions were taken in equimolar quantities, mixed well, and left to stabilize for 30 min at RT. To formulate the oligo-dT-dA delivery system, the solution of polymers in DMSO (2 mg/mL) was added to the oligo-dT-dA solution in deionized water under intensive stirring. The quantities of oligonucleotide and polymers solutions to be mixed were calculated to achieve polymer/oligo-dT-dA mass ratios in a range from 2/1 to 25/1. The mixture was incubated for at least 3 h at room temperature for stabilization of the complexes. The encapsulation efficacy of oligo-dT-dA-loaded delivery systems was assessed by DLS/ELS and agarose gel electrophoresis (Section 2.2.9).

In addition, we used TAMRA-oligo-dT-dA to perform a quantitative analysis of loading. After loading, the freshly prepared dispersions were ultrafiltrated to separate the solution from nanoparticles (10,000× *g*, 4 °C, 20 min) using Amicon Ultra filter tubes with MWCO 30,000 (Merck, Darmstadt, Germany). The filtrate was collected and then analyzed with the use a ThermoScientific Varioscan (Waltham, MA, USA) microplate reader (λ_ex_ = 546 nm, λ_em_ = 579 nm). The amount of free TAMRA-oligo-dT-dA was calculated from the preliminarily built linear calibration plot. The entrapment efficacy (EE) was calculated as the difference between the initial and non-bound amount of nucleic acid.

#### 2.2.6. Preparation and Characterization of Dual-Component Delivery Systems

Dual-component delivery systems containing PTX and oligonucleotide duplex simultaneously were obtained by the same procedure. Firstly, the PTX solution in DMSO (1 mg/mL) was added to the polymer dissolved in DMSO (2 mg/mL), mixed well, and then the obtained solution was dropped into an oligo-dT-dA solution of the desired concentration in deionized water under intensive stirring. After a 3-h incubation of the obtained complexes, DLS/ELS measurements and gel electrophoresis were performed.

#### 2.2.7. PTX Release Study

Paclitaxel release was studied by the dialysis method under model physiological conditions (0.01 M PBS, pH 7.4, T = 37 °C). For this, 500 µL of the solution containing 1 mg of the PTX-loaded system under investigation was placed into a dialysis tube (MWCO 3500). Then, the tube was put into 12 mL PBS (0.01 M, pH 7.4) (external solution) and shaken at 37 °C. At predetermined time intervals, 3 mL of external solution was withdrawn for analysis and 3 mL of fresh pre-warmed PBS was added. Then, the collected samples were freeze dried and the PTX amount was analyzed by reversed-phase HPLC using a previously reported protocol [51]. The analysis was carried out at the Chemical Analysis and Materials Research Centre of SPbU Research Park.

For analysis of the PTX release curves, a number of mathematical dissolution models (zero-order, first-order, Higuchi, Hixson–Crowell, Korsmeyer–Peppas, Baker–Lonsdale, Hopfenberg, Weibull, Gompertz, and Peppas–Sahlin [52]) were applied. The DDSolver add-in for Microsoft Excel was used for mathematical analysis [53].

#### 2.2.8. Oligo-dT-dA Release Study

The in vitro release of TAMRA-oligo-dT-dA from nanoparticles was studied within 7 days. In total, 100 µL of the freshly prepared test formulation was diluted with 300 µL of the release medium (0.01 M Na-phosphate buffer, pH 7.4) and incubated at 37 °C under stirring (300 rpm). After a certain period, the dispersions were ultrafiltrated and filtrates containing free TAMRA-oligo-dT-dA were analyzed as described in Section 2.2.5. The formulations with a determined optimal ratio of polypeptide/oligo-dT-dA were used for this study.

#### 2.2.9. Agarose Gel Electrophoresis

Electrophoresis of the samples was performed in 1.5% gel in 1 M TAE buffer solution, pH 7.6, at an operating voltage of 40 V for 40 min. Agarose gel was prepared by dissolving agarose in 1 M TAE buffer solution, pH 7.6, under heating before an aliquot of EtBr 10 mg/mL was added to the solution so that the resulting EtBr concentration was equal to 0.5 µg/mL. After electrophoresis, gels were imaged using an Amersham Imager 600 Instrument (GE Healthcare, Little Chalfont, UK).

The total volume of each sample for electrophoresis analysis was equal to 20 µL, each sample contained 5 µL of the 4× loading xylene blue and bromophenol blue dye (Evrogen, Moscow, Russia). In order to enable visualization, oligonucleotides labelled with TAMRA fluorescent dye were bound to the oligo-dT-dA duplex. At least 200 ng of oligo-dT-dA was loaded into each gel well in the form of complexes with polypeptides, and free oligo-dT-dA was used as a control sample.

#### 2.2.10. Formulation Stability Study

In order to examine the storage stability of the systems under investigation, polypeptides nanoparticles, their PTX and oligo-dT-dA-loaded complexes, and the dual-component system were obtained and their initial *D_H_* and PDI were measured. Then, samples were stored at 20 °C for 21 days with regular measuring of the *D_H_* and PDI parameters.

The impact of the presence of competing polyanions was examined via the heparin displacement test. For this, polypeptides’ PTX-oligo-dT-dA-loaded complexes were mixed with heparin in water solutions of different concentrations (0.15, 10, and 40 IU). After 30 min of incubation, agarose gel electrophoresis was performed (Section 2.2.9).

#### 2.2.11. Cytotoxicity

In order to evaluate the cytotoxicity of the polypeptide particles, a cell viability assay was performed using HEK 293T, HeLa and A549 cells. Cells were cultivated in Dulbecco’s modified Eagle’s medium containing 10% (*v*/*v*) fetal calf serum (FCS) and 1% gentamicin. A humidified environment at 37 °C and 5% CO_2_ was applied, changing the medium three times per week. In 96-well plates, 4 × 10^3^ cells in 100 μL were seeded per well and left to initiate adhesion at 37 °C in a humidified 5% CO_2_ atmosphere. After 24 h, the culture medium was replaced with the same medium but containing polypeptide particles with different concentrations (from 1 to 250 µg/mL). The percentage of viable cells was determined by the MTT assay after 72 h. For this, 30 µL of MTT solution (5 mg/mL) was added to each well. The plates were incubated for 2 h and then the MTT solution was aspirated and replaced with 100 µL DMSO. Gentle shaking was applied to simplify crystals of formazan dissolution. Finally, the optical density in each well was measured using a fluorescent plate reader (Fluoroscan Ascent, Thermo Fisher Scientific Inc., Waltham, MA, USA) at 570 nm. To measure the background absorbance, the wavelength of 690 nm was used. The relative cell viability (%) was calculated according to the equation:

Cell viability = (*A_sample_* − *A_blanck_*)/(*A_control_* − *A_blanck_*) × 100% (1)

#### 2.2.12. Cytostatic Effect of PTX

The cytostatic effect of paclitaxel in the form of a free drug and PTX-loaded particles was evaluated by a cell viability assay using A549 (human alveolar epithelial adenocarcinoma) cells. Cells were cultivated in Dulbecco’s modified Eagle’s medium containing 10% (*v*/*v*) fetal calf serum (FCS) and 1% gentamicin in a humidified environment at 37 °C and 5% CO_2_. The experiment was carried out as described in Section 2.2.11 but using free PTX or PTX-loaded nanoparticles as test materials. The concentrations of PTX were varied from 1 to 250 ng/mL. Non-linear-curve-fitting/growth/sigmoidal/dose–response-fitting functions (OriginPro 8.6) were used to build dose–response curves for concentration-dependent normalized cell viability data and calculate the half-maximal inhibition concentrations (IC_50_).

#### 2.2.13. Gene Silencing: Flow Cytometry

In order to evaluate siRNA-loaded polypeptides delivery systems for transfection, K562 cells modified with the d2EGFP plasmid (K562/GFP) were used. Cells were cultivated in RPMI-1640 cell culture medium supplemented with 10% FCS, 1 µg/mL puromycin, and 50 µg/mL gentamicin and incubated in a humidified environment at 37 °C and 5% CO_2_. To obtain hybridized siRNA strands, GFP-siRNA-sense and GFP-siRNA-antisense were mixed in a ratio of 1:1 and heated at 90 °C for 1 min. Then, the mixture was left to cool at RT for 30 min.

siRNA-loaded systems were obtained according to the above-described procedure (Section 2.2.5). As a positive control, GenJect-39 and GenJect-40 were used. Herein, 1 µL of either GenJect-39 or GenJect-40 was diluted in 25 µL of full growth medium. In parallel, a corresponding amount of siRNA was diluted in 25 µL of full growth medium. Then, diluted siRNA was mixed with GenJect reagents dropwise in a ratio of 1:1. After gently mixing, complexes were incubated at RT for 30 min.

A total of 3 h before the experiment, 450 µL of K562/GFP cells were seeded on 24-well plates at a density of 1.1 × 10^4^/mL and kept in an incubator. Then, siRNA-loaded polypeptides complexes and GenJect-siRNA control complexes were added to K562/GFP pre-seeded cells. Plated cells were incubated at 37 °C and 5% CO_2_ for 2 days. Afterwards, cells were collected, centrifuged at 500× *g* for 3 min, and resuspended in 200 µL of PBS (pH 7.4). Flow cytometry (Beckman Coulter, Brea, CA, USA) using FITC filter (488 nm excitation, 525/40 BP nm emission) was performed immediately after resuspension.

#### 2.2.14. Gene Silencing: RT-PCR

After complex-formation reagents were added to K562/GFP pre-seeded cells, plated cells were incubated at 37 °C and 5% CO_2_ for 2 days. After incubation was complete, cells were collected, centrifuged at 500× *g* for 3 min, and pellets were resuspended in 800 µL ExtractRNA (Evrogen, St. Petersburg, Russia) and lysed for 5 min. Then, the extraction was performed according to the manufacturer’s instructions.

The RNA concentration and quality of the samples were measured using Implen (Implen, Westlake Village, CA, USA), and 2 µg of RNA from each sample was taken for RT-qPCR. MMLV reverse transcription was conducted according to the instructions of the manufacturer. Then, qPCR was performed using HS-qPCR mixes with SYBR Green I according to the instructions of the manufacturer (Evrogen, St. Petersburg, Russia). As a gene of interest, GFP was chosen. B2M was chosen as a housekeeping gene. As a negative control, RNAse-free water was used with the same primers.

Changes in target genes’ expression were estimated on the base of differences in the quantification cycles ($\Delta C_{q}$) of the control and treated samples using the equation:

(2) $$R=E^{\Delta C_{q}^{\mathrm{housekeeping}\;\mathrm{gene}}-\Delta C_{q}^{\mathrm{target}\;\mathrm{gene}}}$$

#### 2.2.15. Statistical Analysis

All measurements of the polypeptide particles’ characteristics were performed three times. Biological experiments were performed from 3 to 6 times for each kind of tested sample. In all cases, the data are presented as the mean value ± SD. A student’s *t*-test was used to analyze the statistical significance of the results. The results are considered as statistically significant if *p* < 0.05.

## 3. Results and Discussion

### 3.1. Polymer Synthesis and Characterization

**Figure 1:**
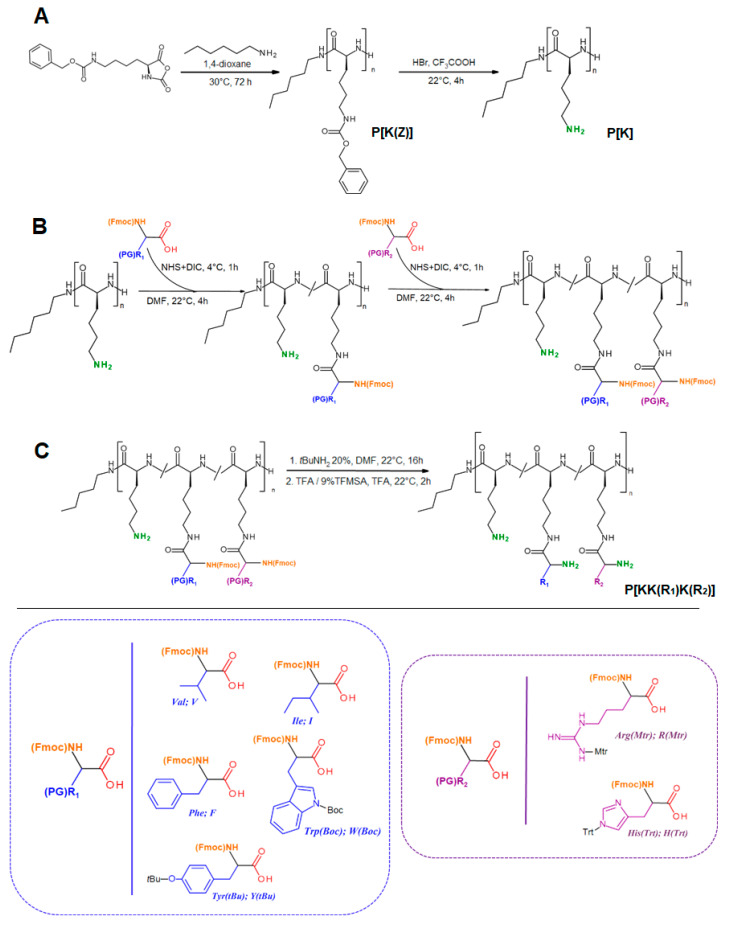
Scheme of the synthesis of cationic amphiphilic polypeptides: (**A**) synthesis of P[K] by ROP; (**B**) post-polymerization modification of P[K] by hydrophobic and basic amino acids; (**C**) deprotection of side chain functional groups.

The synthesis of amphiphilic polypeptides included (i) ring-opening polymerization of Lys(Z) NCA and (ii) post-polymerization modification of the resulting poly(αl-lysine) (P[K]) with hydrophobic and basic amino acids. The scheme of synthesis is presented in Figure 1.

The synthesized Z-protected poly(αl-lysine) (P[K(Z)]) was characterized by static light scattering (SLS) to determine the average molecular weight (*M_w_*). As can be seen from Table 1, the protected polymer (fully soluble in DMF) has an *M_w_* equal to 16,800.

According to ^1^H NMR spectroscopy (Figure S1, Supplementary Materials), the degree of polymerization (*DP*) was equal to 93. The total removal of the Z-group was testified by ^1^H NMR spectroscopy (Figure S1, Supplementary Materials).

The post-polymerization modification of P[K] was performed in two steps. During the first step, the polymer was modified with one of the hydrophobic amino acids from the series: Val, Ile, Tyr, Phe, and Trp. With this aim, the α-Fmoc-protected derivatives of amino acids were utilized. In addition, besides valine and isoleucine, other amino acids were used in the side-chain protected form (Figure 1). The modification was carried out via activation of the carboxylic group of the amino acid derivative to an activated ester followed by the reaction with free ε-amino groups of P[K]. The molar ratio of hydrophobic amino acid to lysine units in the homopolymer was equal to 0.30.

During the second step, the hydrophobized poly-l-lysine was further modified with Arg or His to generate an additional functionality for siRNA binding and delivery. As a result of the strong positive charge of Arg and the pH-sensitivity of His under physiological conditions, they were used in different molar ratios with respect to the Lys-units of hydrophobized copolymers. In particular, this ratio was 0.45 for Arg and 0.60 for His derivatives.

As for the lysine homopolymer, SLS in DMF was also applied to determine the molecular weight of hydrophobized P[K] before its modification with Arg or His. However, *M_w_* determination was only possible for copolymers containing Val, Ile, and Tyr (P[KK(V)], P[KK(I)], and P[KK(Y)], respectively (Table 1). The copolymers containing the most hydrophobic Phe and Trp residues did not provide the true solutions in DMF. As a result of the detection of aggregates up to 60 nm even in organic solvents, the determination of molecular weight by SLS (as well as SEC) was not possible. For the other three copolymers, the hydrodynamic radii of the macromolecules were successfully measured. One can observe an increase in the size of the substituent (from Val to Tyr), followed by an increase in *M_w_*, and simultaneously with a decrease in hydrodynamic radius (Table 1). Although the calculation of *M_w_* for the Phe-containing amphiphilic copolymer was impossible due to the presence of aggregates, a mode corresponding to the dissolved macromolecules with a hydrodynamic radius of 0.8 nm was detected (P[KK(F)], Table 1). Thus, a decrease in the hydrodynamic radius of macromolecules from 2.1 to 0.8 was revealed in the series of amphiphilic copolymers based on poly-L-lysine containing Val, Ile, Tyr, and Phe. The most pronounced densification, observed for Tyr and Phe-containing polypeptides, can be caused by the formation of tightly packed macromolecular coils due to the introduction of aromatic substituents that can additionally provide π–π interactions.

For hydrophobic amino acids, the degree of substitution was calculated both from the ^1^H NMR spectra (Figure S2, Supplementary Materials) and from the data of quantitative HPLC analysis of amino acids produced after total acidic hydrolysis of the amphiphilic polypeptides (Table 2). At the same time, after modification of hydrophobized P[K] with Arg/His, calculation of the copolymer composition was impossible due to the overlapping signals in the ^1^H NMR spectra. For these polymer samples, the composition was determined by quantitative HPLC amino acid analysis of the polypeptide hydrolysates (Table 2). Certain ^1^H NMR spectra of His/Arg protected polypeptides are shown in Figure S3 in the Supplementary Materials.

In all cases, the modification of P[K] with hydrophobic amino acid was less than theoretically specified (30 mol%). Overall, the modification efficacy for hydrophobic amino acids ranged from 60 to 90%. The highest modification efficacy was observed for sterically non-complicated aliphatic amino acids. In the case of modification with Arg, the modification efficacy was around 50–88% and was higher for amphiphilic copolymers containing fewer hydrophobic amino acids (Val, Ile, Tyr). In turn, the efficacy for the modification of poly-l-lysine copolymers with His was around 70% in all cases. The modification efficiency obtained in this work is consistent with recently published data on the modification of poly(αl-glutamic acid) with various amino acids and d-glucosamine [51].

In addition, Table 2 also represents the approximate values of *M_n_* calculated basing on degree of polymerization of the main chain, namely P[K], and averaged content of the amino acids introduced during modification. As can be seen, the *Mn* values for the obtained polypeptides were very close and varied in the range of 18.5–20.6 kDa.

### 3.2. Preparation and Characterization of Single and Dual-Component Formulations

The synthesized copolymers were used to load PTX and double-stranded nucleic acid both individually and simultaneously. PTX is an effective cytostatic drug and is included in the first-line treatment of breast and ovarian cancer, as well as carcinomas of the lung, prostate, and brain [54,55]. PTX’s high hydrophobicity is the reason for its poor aqueous solubility and bioavailability. At the same time, PTX nanoformulations allow the improvement of patient outcomes in cancer chemotherapy [56,57]. The loading of hydrophobic PTX was achieved as a result of the hydrophobic interactions between the drug and the hydrophobic amino acid units of the polypeptide chain.

In contrast to cytostatic drugs, which differ significantly in their structure and physicochemical properties (hydrophilic/hydrophobic balance, chemical functionality, charge, solubility, etc.), small double-stranded nucleic acids have comparable physicochemical properties, which are mainly determined by their negative charge and high solubility in aqueous media. The key factor for such molecules is their length, which impacts the total negative charge in the chain, and consequently, the strength of binding to the polycation. Taking this into account, the dT-dA oligonucleotide duplex (oligo-dT-dA, 23 b.p.) was used as a stable physicochemical model of siRNA to evaluate the binding of short double-stranded nucleic acids with the synthesized polypeptides. Nucleic acid retention was provided by a polyelectrolyte interaction between the cationic fragments of the polypeptide and the anionic nucleic acid.

Both hydrophobic and polyelectrolyte interactions were involved in the production of particulate formulation under simultaneous loading of PTX and small double-stranded nucleic acid.

#### 3.2.1. PTX-Loaded Systems

The loading of PTX was performed under varying initial amounts of the drug. For this purpose, two series of polypeptides containing aliphatic and aromatic hydrophobic moieties, namely P[KK(I)K(R)], P[KK(I)K(H)], P[KK(Y)K(R)], and P[KK(Y)K(H)], were used for the study. The PTX amount taken for loading was varied from 25 to 150 μg/mg of polymer. In all cases, the loading efficacy was 99–98%.

The resulting high encapsulation efficacy is consistent with our previous results on the encapsulation of PTX into nanoparticles based on random amphiphilic polypeptides obtained by the ROP NCA [48,58]. In a recent study, a high efficacy of PTX encapsulation was also revealed for amphiphilic polypeptides produced by post-polymerization modification of the glutamic acid homopolymer with various hydrophobic amino acids, l-histidine, and d-glucosamine [51]. In that case, increasing the initial amount of PTX from 25 to 150 μg/mg of polymer provided an encapsulation efficacy from 82 to 99%. The reduced loading at an initial PTX amount of 150 μg/mg of polymer for this group of polypeptides was explained by the lower content of hydrophobic amino acids (6–9 mol%) compared to the polypeptides in this study (16–25 mol%).

**Figure 2:**
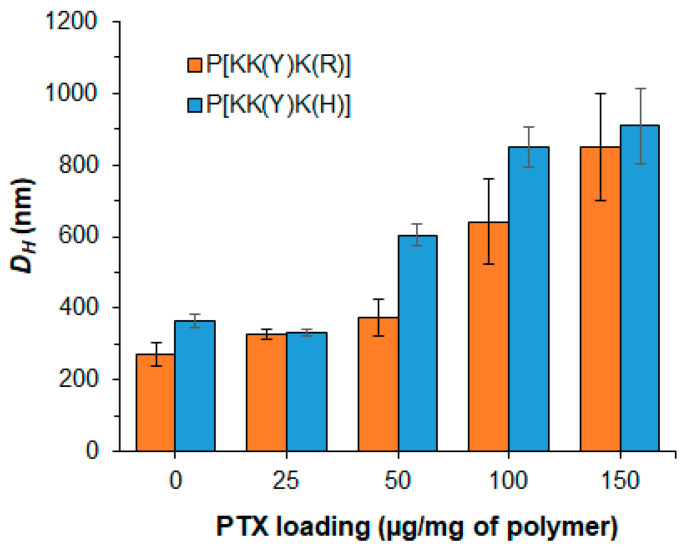
Effect of PTX loading into polypeptide nanoparticles on the hydrodynamic diameters of the delivery systems.

The effect of PTX loading on the hydrodynamic diameter of the delivery systems was evaluated by DLS in the loading range of 25–150 μg PTX per mg of polymer (Figure 2). It was found that for Tyr-containing polypeptide particles (P[KK(Y)K(R)] and P[KK(Y)K(H)]), the loading of 25 μg PTX per mg of polymer had no effect on the hydrodynamic diameter of the polymer particles. The same result was also observed for P[KK(Y)K(R)] when 50 μg PTX/mg of polymer was loaded. Despite retaining a high encapsulation efficacy, an increase in loading to 100–150 μg PTX/mg of polymer was accompanied by a significant increase in *D_H_* for both P[KK(Y)K(R)] and P[KK(Y)K(H)].

#### 3.2.2. Nucleic-Acid-Loaded Systems

The formation of polyelectrolyte complexes between polypeptide and oligo-dT-dA was performed by dropwise addition of an aliquot of the polymer solution in DMSO to an aqueous solution containing a predetermined amount of oligo-dT-dA under vigorous stirring. The polypeptide/oligo-dT-dA ratio was varied from 20 to 2.

**Figure 3:**
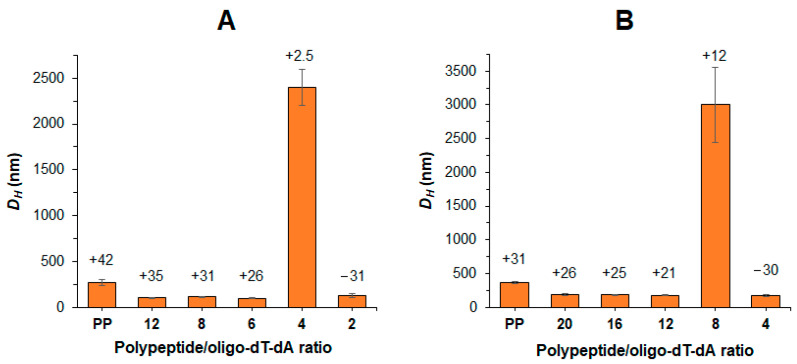
Effect of polypeptide/oligo-dT-dA ratio on *D_H_* and ζ-potential (above the bars) for the complexes based on P[KK(Y)K(R)] (**A**) and P[KK(Y)K(H)] (**B**) (PP is non-loaded polypeptide particles).

After the stabilization of the complexes, their characteristics were analyzed by DLS. As expected, the formation of polypeptide/oligo-dT-dA complexes was accompanied by a decrease in *D_H_* compared to non-loaded polypeptide particles (PP) (Figure 3). Since Arg-containing polypeptides have a stronger positive charge than His-containing ones, a higher loading of oligo-dT-dA into Arg-bearing polypeptides was observed without recharging the delivery system surface (Figure 3). Complexes of Arg-containing polypeptides with oligo-dT-dA could retain their size up to a ratio of 6, whereas complexes of His-containing polypeptides only retained their size up to 12.

**Figure 4:**
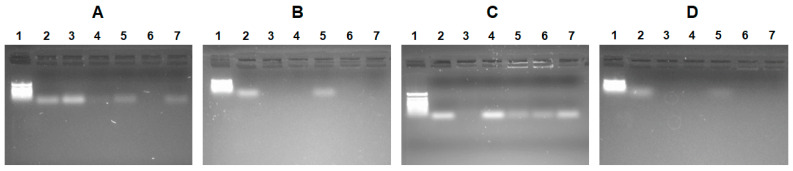
Agarose gel electrophorese images: Arg-containing polypeptide/oligo-dT-dA ratio = 8 (**A**), Arg-containing polypeptide/oligo-dT-dA ratio = 12 (**B**), His-containing polypeptide/oligo-dT-dA ratio = 12 (**C**), His-containing polypeptide/oligo-dT-dA ratio = 16 (**D**). Lanes: 1—markers; 2—free oligo-dT-dA; (**A**,**B**) 3—P[KK(F)K(R)]@oligo-dT-dA; 4—P[KK(V)K(R)]@oligo-dT-dA; 5—P[KK(I)K(R)]@oligo-dT-dA; 6—P[KK(Y)K(R)]@oligo-dT-dA; 7—P[KK(W)K(R)]@oligo-dT-dA; (**C**,**D**) 3—P[KK(F)K(H)]@oligo-dT-dA; 4—P[KK(V)K(H)]@oligo-dT-dA; 5—P[KK(I)K(H)]@oligo-dT-dA; 6—P[KK(Y)K(H)]@oligo-dT-dA; 7—P[KK(W)K(H)]@oligo-dT-dA.

In addition to the size of the complex, another important issue is the effective binding of the nucleic acid by the polypeptide. In order to assess the efficiency of oligo-dT-dA binding by polypeptides, agarose gel electrophoresis was applied (Figure 4). During the first step, polypeptide/oligo-dT-dA ratios of 8 and 12 were examined for Arg- and His-containing polypeptides, respectively (Figure 4A,C). At these ratios, effective binding was observed for only two Arg-containing polypeptides (P[KK(V)K(R)] and P[KK(Y)K(R)]) and one His-containing polymer (P[KK(F)K(H)]). Increasing of the polypeptide/oligo-dT-dA ratios to 12 and 16 for Arg- and His-containing polypeptides, respectively, was followed by an improvement in binding for all polypeptides, except both Ile-containing ones (P[KK(I)K(R)] and P[KK(I)K(H)]) (Figure 4B,D). For these, the optimal ratios to ensure efficient binding were 16 and 20 for P[KK(I)K(R)] and P[KK(I)K(H)], respectively. At the optimal ratios, the oligo-dT-dA load was ≥99%.

For comparison, Qiu et al. reported the development of a series of PEGylated lysine–leucine-based peptides with a molecular weight of 3–4 kDa for siRNA delivery. They found that the optimal carrier/siRNA (*w*/*w*) ratio was 10 or more [59]. The same mass ratio was found to be effective for the total binding of siRNA by lysine–arginine-containing peptides modified with a fatty acid tail (C12–C20) [60]. Svirina et al. developed two cationic cell-penetrating peptides, one of which could effectively bind siRNA at mass ratios of 10 and 20, and the other at 20 or higher [61]. Thus, the effective ratios established in this work are consistent with the results obtained by other research groups.

#### 3.2.3. Dual-Component Systems

**Figure 5:**
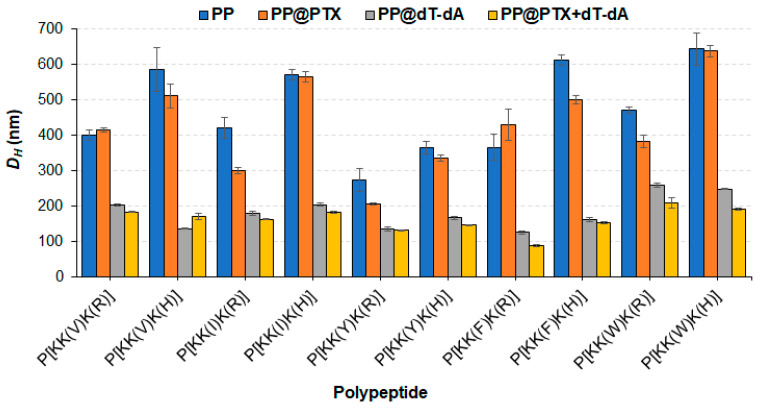
Comparison of hydrodynamic dimeters of empty polypeptide particles (PP), single-laded particles containing PTX (PP@PTX) or oligo-dT-dA (PP@oligo-dT-dA), as well as dual-component systems (PP@PTX + oligo-dT-dA). The dual-component delivery systems were prepared using 25 μg of PTX per mg of polymer and at the optimal polypeptide/oligo-dT-dA ratios: 12 for Arg-containing polypeptides and 16 for His-containing ones. For Ile-containing polypeptides, the polymer/oligo-dT-dA ratios were 16 and 20 for P[KK(I)K(R)] and P[KK(I)K(H)], respectively.

The dual-component delivery systems containing PTX and oligo-dT-dA were prepared similarly to the oligo-dT-dA formulations with the only difference being that the PTX and polymer solution in DMSO was added dropwise to an aqueous oligo-dT-dA solution under vigorous stirring. The hydrodynamic diameters of the empty polypeptide particles (PP), particles loaded with PTX (PP@PTX) or oligo-dT-dA (PP@oligo-dT-dA), as well as dual-component delivery systems (PP@PTX + oligo-dT-dA) were evaluated by DLS (Figure 5).

Analysis of the data clearly shows that the empty nanoparticles are quite loose with a *D_H_* of 300–600 nm depending on the polymer. PTX loading has a weak effect on the hydrodynamic diameter of the nanoparticles. In general, His-containing polymers formed larger particles than Arg-containing ones. This can probably be explained by the greater repulsion of positively charged Arg-containing polymer chains, which contributes to the formation of initially smaller particles in aqueous medium. In our recent work on the post-polymerization modification of poly(glutamic acid) with cationic amino acids, hydrophobic amino acids, L-histidine, and d-glucosamine, the synthesized copolymers formed nanoparticles of 200–400 nm in size, although they contained hydrophobic amino acid in a lower amount (6–9 mol%) [51]. The smaller size in that case was explained by the simultaneous presence of positively and negatively charged amino acids in the polypeptide which favored the compaction of the polymer particle due to polyelectrolyte interaction. Indeed, the formation of polypeptide complexes based on amphiphilic modified polylysine and nucleic acid contributed to a significant compaction of polymer particles. Apart from Trp-containing polymers, other polymers in complex with oligo-dT-dA form particles with sizes ranging from 120 to 205 nm. The largest nanoparticles (230 and 250 nm) were formed by polypeptides containing Trp, possessing the largest hydrophobic substituent in the side chain.

In turn, the two-component delivery systems formed more compact nanoparticles of 90 to 200 nm compared to single-component formulations. Moreover, the encapsulation of PTX and nucleic acid had a positive effect on the formation of narrowly dispersed particles.

According to the literature data, the hydrodynamic diameter of peptide-based complexes with siRNA ranges from 70 to 200 nm [62,63,64], but complexes of 400 and 600 nm have also been reported [59,65]. In this respect, the polypeptide and siRNA complexes obtained in this work are comparable with the majority of other peptide/siRNA complexes.

**Figure 6:**
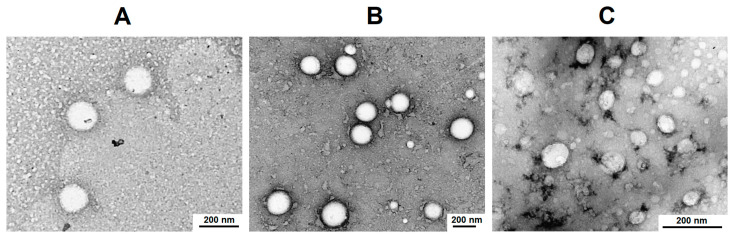
TEM images of empty PP (**A**), PP@PTX (**B**), and PP@PTX + oligo-dT-dA (**C**) based on the P[KK(Y)K(R)] polypeptide. Scale bar is 200 nm; staining with uranyl acetate. In (**B**,**C**), the PTX load was 50 μg/mg of polymer; the ratio of polypeptide/oligo-dT-dA in (**C**) was 12 (*w*/*w*).

In addition, the morphology of the empty and PTX-loaded systems was evaluated by transmission electron microscopy (TEM) for P[KK(Y)K(R)]-based particles (Figure 6). In all cases, polypeptide particles represent nanospheres. The average diameter of empty PP, PP@PTX, and PP@PTX+oligo-dT-dA systems based on the P[KK(Y)K(R)] was 156 ± 30, 153 ± 57, and 75 ± 25 nm, respectively. In all cases, the average diameter determined by TEM was lower than their hydrodynamic diameter. For the encapsulated forms, the average diameter in the dry state (TEM) was 30% less than the corresponding hydrodynamic diameter (DLS), while for the empty nanoparticles it was twice as low. This supports the assumption made above that the empty nanoparticles are quite loose due to electrostatic repulsion between the polymer chains within the particle. Compression during dehydration is known to occur for soft nanomaterials [58,66,67]. PTX encapsulation decreases the hydrodynamic diameter, while the average diameter in the dry state remains unchanged. In turn, the loading of nucleic acid together with PTX significantly reduces both the hydrodynamic diameter and the average diameter in the dry state.

#### 3.2.4. Stability of Formulations

**Figure 7:**
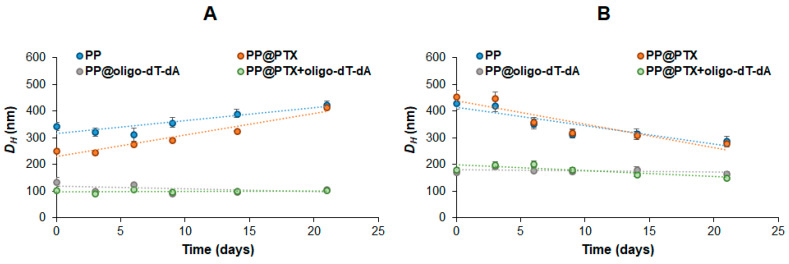
Storage stability of empty nanoparticles, as well as single- and dual-component systems based on P[KK(Y)K(R)] (**A**) and P[KK(Y)K(H)] (**B**) polypeptides, within 3 weeks at room temperature (20 °C, pH 7.4).

The storage stability of empty polypeptide particles and single- and dual-component formulations was investigated by monitoring the hydrodynamic diameter using DLS (Figure 7). All test systems were incubated at room temperature, maintained at 20 °C, and the *D_H_* was measured at set intervals within 3 weeks. Empty and PTX-loaded polypeptide particles showed the same trend: the hydrodynamic diameter of Arg-containing polymers increased by 15–30%, while that of His-containing polypeptides decreased by about 50%. This can be explained by the reorganization of the polypeptide chains and, as a consequence, particles over time. At the same time, both oligo-dT-dA-loaded and dual-component polypeptide particles were stable and did not change in size over time. This fact may indicate a dense packing of these formulations stabilized simultaneously by hydrophobic and polyelectrolyte interactions.

**Figure 8:**
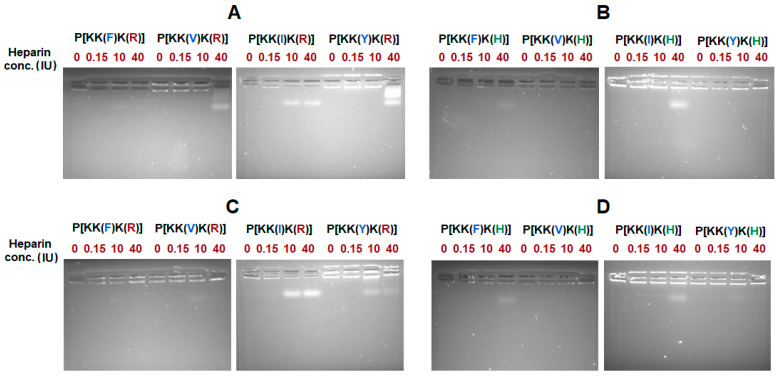
Heparin displacement test (agarose gel electrophoresis): (**A**) PP@oligo-dT-dA based on Arg-containing polypeptides; (**B**) PP@oligo-dT-dA based on His-containing polypeptides; (**C**) PP@PTX+oligo-dT-dA based on Arg-containing polypeptides; (**D**) PP@PTX+oligo-dT-dA based on His-containing polypeptides. The concentration of heparin was varied from 0 to 40 IU. In the case of Arg-containing polypeptides besides P[KK(I)K(R)], the polypeptide/oligo-dT-dA ratio was 12; for P[KK(I)K(R)], it was 16. In the case of His-containing polypeptides besides P[KK(I)K(H)], the polypeptide/oligo-dT-dA ratio was 16; for P[KK(I)K(H)], it was 20.

In addition, the stability of single and dual-component systems was explored in the presence of heparin which is a strong polyanion under a wide pH range. The concentrations of heparin were varied from 0.15 IU, which is the physiological concentration in the extracellular matrix, to 40 IU. As can be seen from Figure 8, among the Arg-containing polypeptides, the strongest retention of nucleic acid was observed when Phe was used as a hydrophobic moiety (P[KK(F)K(R)]). In this case, no displacement was detected for either the single or dual-component systems at any concentration tested (Figure 8A,C). In turn, for other hydrophobic amino acids, the displacement of oligo-dT-dA by heparin was revealed for all other Arg-containing systems (Figure 8A). The loading of PTX to the system stabilized the Val- and Tyr-containing nanoparticles, increasing the stability of these delivery systems, and did not affect the Ile-containing system (Figure 8A,C).

For His-containing polypeptides, the displacement of oligo-dT-dA was observed for all formulations at a heparin concentration of 40 IU except systems containing Val and Tyr as hydrophobic amino acids (Figure 8B,D).

### 3.3. Drug Release and Mechanism Study

#### 3.3.1. PTX

**Figure 9:**
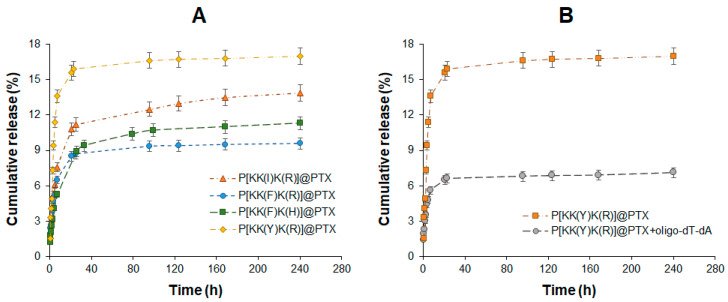
PTX release profiles from single (**A**) and double (**B**) component formulations (0.01 M PBS, pH 7.4, 37 °C).

Considering that PTX loading and retention in the polypeptide particle are controlled by hydrophobic interactions, the lipophilicity of the hydrophobic amino acid should influence the PTX release rate. According to published data [68,69], the lipophilicity of the used amino acids is increased in the following order: Y < V < I < F < W (Table S1, Supplementary Materials). Thus, tyrosine is the least lipophilic amino acid in the series, while tryptophan is the most lipophilic one. The highest lipophilicity of tryptophan and largest size-chain substituent contribute to the formation of the largest nanoparticles. Given this, Trp-containing nanoparticles were excluded from the study as suboptimal. The PTX formulations based on the polypeptides containing the less lipophilic (Y), intermediate (I), and most lipophilic after tryptophan (F) amino acid were selected for the release study. In addition, the release from Arg- and His-containing polypeptide delivery systems was compared using the Phe-containing series (P[KK(F)K(R)] and P[KK(F)K(H)]). The release of PTX was studied using model conditions (0.01 M PBS, pH 7.4, 37 °C). The profiles of the cumulative release are shown in Figure 9A.

As may be supposed, the release rate was in agreement with the lipophilicity of the hydrophobic amino acid. The Tyr-containing system demonstrated the fastest PTX release, while the Phe-containing one indicated the slowest release. In particular, the release of PTX after two weeks was 16.8 ± 0.8, 13.9 ± 0.6, and 9.6 ± 0.5% for P[KK(Y)K(R)], P[KK(I)K(R)], and P[KK(F)K(R)], respectively.

A similar release rate was observed by Hou et al. who studied the release from nanoparticles formed from amphiphilic peptides specific to colorectal cancer and PTX. In particular, 8 and 9% PTX release was detected after 50 h of incubation of the delivery systems in buffer and culture medium [70], respectively. Li et al. reported the release of PTX by 37% for 70 h from an amphiphilic delivery system combining PEG-*b*-PLA and a PAMAM dendrimer (G12) [71]. Thus, the nature and structure of the delivery system have a significant influence on the rate of release.

In order to study the release of PTX from a dual-component formulation, the P[KK(Y)K(R)] polypeptide was used as a delivery system. The Tyr-containing polypeptide was selected due to having the highest release rate from a single-component system observed for this polymer. This fact allowed us to expect better detection of the effect of a small double-stranded nucleic acid complexed with the polymeric carrier. Indeed, PTX release from a delivery system containing PTX and oligo-dT-dA (physicochemical siRNA model) was significantly decelerated (Figure 9B). An approximately two-fold decrease in PTX release was detected within two weeks from such a system (7.2% from the dual-drug system versus 16.8% from the single-drug system). This fact is explained by the additional polyelectrolyte compaction of the two-component delivery system compared to the PTX-loaded one. This is in agreement with the hydrodynamic diameter of such systems: 205 nm for P[KK(Y)K(R)]@PTX and 130 nm for P[KK(Y)K(R)]@PTX+oligo-dT-dA. The denser packing of polymer chains, in turn, slows down the diffusion of PTX from polypeptide particles.

The PTX release profiles obtained in this study were analyzed with the application of standard mathematical models in order to ascertain the kinetic peculiarities of the dissolution mechanism [72]. It is well known that drug dissolution from polymer-based formulations can be controlled by diffusion or affected by polymer relaxation. Approximation with different mathematical models was applied during the first 7 h and during 240 h of release (Table 3). One can observe that the release during the first 7 h is very nicely approximated with nearly all models. The correlation coefficient is close to 1 in these cases. However, this is not the case for whole release profiles approximations, which are characterized by coefficients significantly below 1. The reason for this is likely the different impact of diffusion and polymer relaxation on the early and late stages of release. Poor fitting with the zero-order model shows that release is dependent on the drug concentration. Notably, the Higuchi and Baker–Lonsdale models give better approximation results than the Hixson–Crowell and Hopfenberg models. This might be explained by the greater effect of diffusion on the release of the drug, in comparison with dissolution or particle degradation [73].

Figure S4 (Supplementary Materials) depicts the best-fitting models. The release profiles were best-approximated with Korsmeyer–Peppas, Gomperz, Peppas–Sahlin, and Weibull models (Table 3). According to n parameters evaluated from the Korsmeyer–Peppas model, the early stage of release (n > 0.5) is affected by polymer relaxation, which could be a swelling of particles. At the same time, the whole curve approximation gives values of the n parameter, which are around 0.2. Thus, one can consider Fickian diffusion as the main mechanism of PTX release from the prepared particulate formulations.

The Gomperz model is usually useful for the description of soluble drug release with an intermediate rate. The relatively good approximation of the 240-h release profile with this model allows us to ascertain the obtained particulate formulations to such variants of controlled-release systems. The α parameter describes the amount of unreleased drug at time t = 1, and this parameter’s values are very close for all systems (see Table 3, values for 240 h of release). At the same time, the β parameter describes the rate of dissolution, whose values are equal (0.247 and 0.248) for P[KK(I)K(R)] and P[KK(F)K(H)], being a bit smaller (0.181) for P[KK(F)K(R)].

The Peppas–Sahlin model appears to be the best model for the approximation of the obtained experimental release profiles. This model takes into account the contribution of both diffusion and relaxation and allows for the evaluation of the effect of these processes by calculation of *k*_1_ and *k*_2_, which are constants showing the impact of diffusion and relaxation, correspondingly. The obtained results are in good correlation with the n parameter values from the Korsemeyr–Peppas model. During the first 7 h of release, the *k*_1_ and *k*_2_ constants have comparable values, while for 240 h of release, the curve approximation *k*_1_ is substantially greater than *k*_2_. This reveals the great role of diffusion in the release of PTX from the obtained nanoparticles.

The Weibull model also showed a good fit for our release data. However, being an empirical model, the parameters in the equations do not describe the mechanism of drug release. The obtained *α* and *β* parameters only reflect the timescale and the shape of the release curve. When *β* is below one, the release curve could be considered as parabolic, which is typical for biphasic release kinetics [74].

Thus, it could be concluded that the release kinetics of PTX from the obtained particulate formulations are generally governed by diffusion, which is greatly affected by polymer relaxation in the first 7 h and then becomes much slower than polymer relaxation (Fickian diffusion).

**Figure 10:**
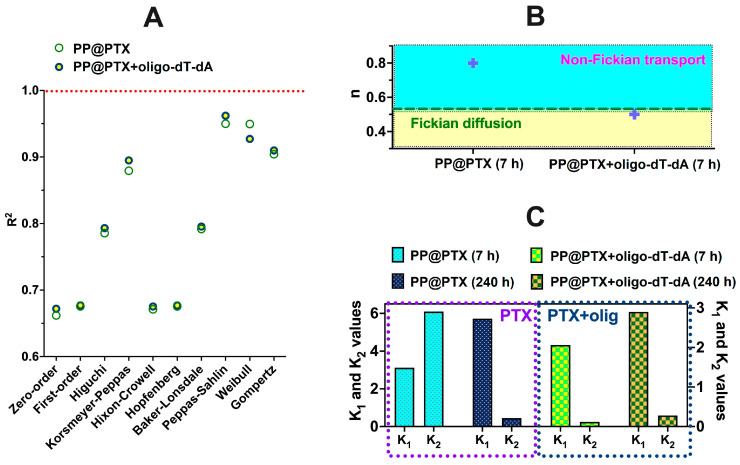
Effect of co-encapsulation of PTX and oligo-dT-dA on the mechanism of PTX release from P[KK(Y)K(R)]-based delivery systems, evaluated with the application of various mathematical modeling: (**A**) comparison of correlation coefficients of the regressions obtained with different models for the release of PTX from single- and dual-component systems during 240 h; (**B**) effect of oligo-dT-dA co-encapsulation on the n parameter evaluated from the Korsmeyer–Peppas model; (**C**) results obtained by application of the Peppas–Sahlin model, where *K*_1_ is the impact of diffusion and *K*_2_ is the impact of relaxation on the release mechanism.

In addition, the effect of oligo-dT-dA on the mechanism of PTX release was assessed. The application of mathematical dissolution models showed that, similar to the results shown above, PTX release alone or from formulations with oligo-dT-dA is better fitted by diffusion-based models than with dissolution or degradation-based ones (Figure 10A). However, the mechanism of this diffusion is different as is evident from results obtained with the Korsmeyer–Peppas model application (Figure 10B). The *n* parameter value for the PTX release alone during the first 7 h of release equals 0.8, which corresponds to the non-Fickian diffusion of the drug. In such a regime, the relaxation and diffusion rates are comparable. In the case of PTX release from the particles with co-encapsulated siRNA in a physicochemical model, the n parameter was about 0.5, which shows that the release of PTX is governed by Fickian diffusion; this means that diffusion is the rate-limiting process. These results are in line with the data obtained from the Peppas–Sahlin model application (Figure 10C). The k_1_ and k_2_ ratio shows the impact of diffusion and relaxation, correspondingly, onto release mechanism. One can observe that in the case of PTX release within the first 7 h, the *k*_1_ and *k*_2_ values possess a similar order of magnitude. The process is affected both by diffusion and polymer relaxation. The whole 240-h release profile modelling according to Peppas–Sahlin showed greater k_1_ values, which reveals the most effect of diffusion in the overall release process. However, this is not so for PTX release from particles with oligo-dT-dA. In this case, the release of PTX is affected more by diffusion (*k*_1_) than by relaxation (*k*_2_) during both the first 7 h of release and also throughout the whole 240 h of the studied release process. The observed effect of oligo-dT-dA on the mechanism of PTX release is most probably due to electrostatic cross-linking of positively charged moieties within the particles by the oligomeric nucleic acid, which results in a denser polymeric matrix of the particles and decelerates the drug diffusion on all stages of release process.

Overall, there is no visible difference in mechanisms for the PTX release from single-drug systems based on P[KK(F)K(R)], P[KK(I)K(R)], P[KK(Y)K(R)], and P[KK(F)K(H)]. However, the addition of the nucleic acid affected both the rate and mechanism of PTX release.

#### 3.3.2. Oligo-dT-dA

The study of the oligo-dT-dA release from single- and dual-component systems based on P[KK(Y)K(R)] and P[KK(Y)K(H)] polypeptide was carried out under the same conditions as for PTX. The monitoring of oligo-dT-dA release over a week revealed a practical absence of free nucleic acid under model conditions (0.1 M PB, 37 °C). In all cases, oligo-dT-dA release did not exceed 1% after one week of incubation. Presumably, the release requires an interaction with a competing polyanion to displace the nucleic acid from the complex with the polypeptide. The ability of a small double-stranded nucleic acid to be displaced by a competing polyanion was demonstrated above (Section 3.2.4) in the heparin displacement test.

### 3.4. Biological Evaluation

#### 3.4.1. Cytotoxicity

The cytotoxicity of the developed polypeptide particles was examined by MTT-assay for 72 h using normal (HEK 293T) and two cancer (HeLa, A549) cell lines. The results regarding the viability of the cells in the range of concentrations from 1 to 250 μg/mL can be found in Figure S5 (Supplementary Materials). The summarized values of the half-maximal inhibitory concentration (IC_50_) are presented in Table 4. It was found that all systems were less toxic to normal cells and were significantly more toxic to cancer cells. This effect is known [75]. For example, earlier it was reported that the IC_50_ for ε-poly(l-Lysine) incubated with L929 cells (normal mouse fibroblast cells) was about 8 mg/mL [75,76]. In turn, a reduction in normal L-02 cells’ (human fetal hepatocytes) viability by up to 40% was observed for ε-poly(l-Lysine) at a concentration equal to 64 μg/mL [77]. At the same time, in the HepG2 cell line (human liver cancer cells), the IC_50_ after 24 and 48 h of incubation was 13.5 and 8.6 μg/mL, respectively [77]. Thus, it can be deduced that the IC_50_ value significantly depends on the cell phenotype. In our case, cytotoxicity for cancer cells was more pronounced for A549 compared to HeLa cells.

A comparison of the cytotoxicity of Arg- and His-containing polypeptides allowed for the conclusion that cytotoxicity was lower for the His-containing polypeptide particles. This result is consistent with previously published data. For instance, Gorzkiewicz et al. studied the cytotoxicity of Lys-, Arg-, and His-containing peptide dendrimers of the third generation and found that the Lys-containing dendrimer was the most toxic (IC50 ≤ 3.5 μM), while the His-containing dendrimer showed lower cytotoxicity (IC50 ~22.2–34.5 μM) to human leukemia monocytic cells (THP1 and U-937) [78].

#### 3.4.2. Inhibitory Effect of PTX

The A549 cell line was selected to study the inhibitory effect of PTX formulations. As a positive control, free PTX (stock solution in DMSO) was examined together with tested samples. According to published data, the IC_50_ for free PTX ranges between 1.1 and 8.5 ng/mL, depending on cell type [79,80,81]. In our case, the IC_50_ value determined for free PTX was 5.1 ± 1.9 ng/mL (Table 5), which is consistent with literature data.

The IC_50_ values determined for the PTX polypeptide-based formulations under study are summarized in Table 5. Certain dose–response curves can also be found in Figure S6 (Supplementary Materials). The IC_50_ values established for PTX polypeptide-based delivery systems were very close to each other (about 4.5–6.2 ng/mL), which is probably due to their similar nature and structure of nanoparticles. Moreover, the obtained formulations showed an IC_50_ close to free PTX, indicating that the required amount of drug was released during the testing period (72 h). In general, the results obtained in this study are similar to those of the previously reported PTX delivery systems based on glycopolymers [48], other polypeptide-based delivery systems [51], or PEGylated phospholipid microparticles [82].

#### 3.4.3. Gene Silencing

GFP-expressing cells represent a convenient model to study siRNA-mediated gene silencing because of the ease in direct detection of a biological effect [83,84]. In our case, a human myelogenous leukemia cell line expressing GFP (K562/GFP) was used for evaluation of the effectiveness of intracellular siRNA delivery and GFP gene silencing. With this aim, the anti-GFP siRNA delivery systems were prepared using Arg-bearing (P[KK(X)K(R)]) polypeptides containing hydrophobic amino acids with different lipophilicity, e.g., X = Tyr(Y), Val(V), Ile(I), or Phe(F). In addition to the Arg-containing polypeptide P[KK(Y)K(R)], its His analog (P[KK(Y)K(H)]) was also involved for comparison. Two commercial transfecting agents, GenJect-39 and GenJect-40, were used as positive controls in the study. Non-treated cells were considered as a negative control.

**Figure 11:**
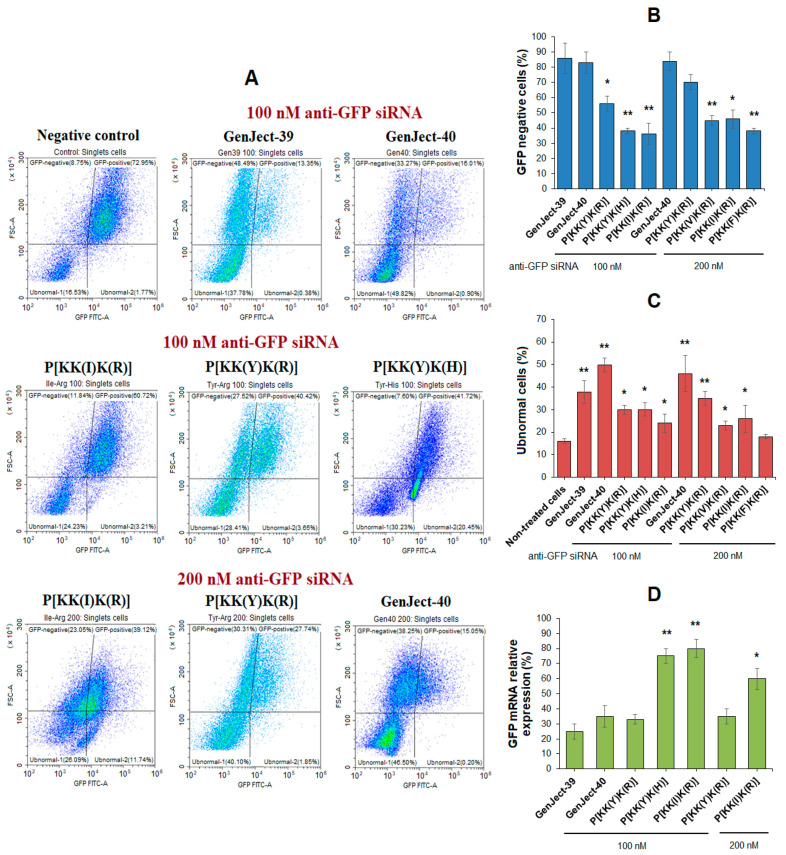
GFP silencing in K562/GFP cells after transfection with PP@anti-GFP siRNA (48 h): (**A**) the results of analysis by flow cytometry; (**B**) total GFP knockdown (flow cytometry); (**C**) ubnormal cells (flow cytometry); (**D**) GFP mRNA expression (RT PCR). The amount of anti-GFP siRNA used for the study was 100 and 200 nM. The polypeptide/siRNA ratio was 12 for Arg-containing polypeptides and 16 for His-containing ones. Complexes of GenJect-39 and GenJect-40 with anti-GFP siRNA were prepared according to the protocol of the manufacturer. The differences with the positive control (GenJect-39/GenJect-40) (**B**,**D**) and negative control (non-treated cells) (**C**) were significant with *p* < 0.05 (*) and *p* < 0.005 (**).

First, the effectiveness of GFP gene silencing was analyzed by flow cytometry after 48 h coincubation of the anti-GFP siRNA delivery systems (100 nM siRNA) with K562/GFP cells (Figure 11A,B). Both commercial transfecting agents showed the highest gene silencing (83–85%). Among the tested polypeptide systems, P[KK(Y)K(R)]@siRNA provided the best silencing of the GFP gene (56%), while the His-containing analog was less effective (38%). The lowest efficiency was observed for the Ile-containing polypeptide (36%). Increasing the siRNA content from 100 to 200 nM favored the growth of transfection efficacy (Figure 11B). In this case, the percentage of GFP-negative cells for P[KK(Y)K(R)]@siRNA system was increased up to 70%, while for GenJect-40@siRNA it remained at the same level (84%) (Figure 11B). A comparison of GFP silencing efficacy for polypeptides differing in hydrophobic amino acids revealed a tendency for decreasing transfection with increasing lipophilicity of the hydrophobic amino acid. Thus, the siRNA-mediated GFP silencing with the Tyr-containing polypeptide system (P[KK(Y)K(R)]) was the highest (70%), while transfection with the Phe-containing polypeptide system (P[KK(F)K(R)]) was the lowest (38%). Most likely, this fact can be explained by the higher intraparticle stabilization due to hydrophobic interactions provided by the more lipophilic amino acid. This, in turn, may affect nucleic-acid release inside the cell due to competitive displacement. The assumption made is supported by the results of the heparin displacement test (Figure 8A,C), from which no oligo-dT-dA release was detected for the P[KK(F)K(R)] delivery system even at the maximum heparin concentration (40 IU). Meanwhile, in the case of the Tyr-containing polypeptide (P[KK(Y)K(R)]), displacement at the same concentration was observed (Figure 8A,C).

In addition, the percentage of ubnormal cells was analyzed by flow cytometry after the transfection experiment for tested and control systems (Figure 11A,C). The comparison of the percentage of ubnormal cells for commercial carriers and polypeptides using 100 nM anti-GFP siRNA revealed a lower percentage of ubnormal cells for all polypeptides. Increasing the amount of anti-GFP siRNA required the use of more polypeptide (while maintaining a constant ratio between them), which in turn was accompanied by an increase in the number of ubnormal cells. In that case, the percentage of ubnormal cells for P[KK(Y)K(R)] approached that of commercial transfecting agents (Figure 11C).

Alternatively, the effectiveness of GFP gene silencing was analyzed by RT PCR after 48 h coincubation of the anti-GFP siRNA delivery systems with K562/GFP cells. Figure 11D shows the relative expression of GFP mRNA when different transfecting agents were applied. In this case, the most effective polypeptide system was P[KK(Y)K(R)], whose efficiency in the reduction in mRNA expression was comparable to commercially available transfecting agents (about 65–75% reduction).

Thus, the developed polypeptide systems were capable of delivering siRNA inside the cells and providing gene silencing, but the P[KK(Y)K(R)]-based system was found to be the most effective. Moreover, in comparison with commercially available transfecting agents, it demonstrated moderate cytotoxicity. At an siRNA concentration of 200 nM, P[KK(Y)K(R)] showed transfection close to GenJect agents but with a lower percentage of ubnormal cells. Furthermore, the developed polypeptide system is fully biodegradable to nontoxic metabolites and can efficiently capture hydrophobic drugs simultaneously with siRNA for their joint delivery.

## 4. Conclusions

In this study, a series of amphiphilic polypeptides with a molecular weight of about 20 kDa was synthesized by a combination of ring-opening polymerization and post-polymerization modification. The main chain of the polypeptides consisted of lysine units, while the side chains contained arginine/histidine and hydrophobic amino acid residues. The particles formed by the polypeptides had a fairly large size (*D_H_* in the range of 400–600 nm) and did not compact after PTX loading. In turn, the loading of short double-stranded nucleic acid significantly contributed to the size reduction (down to 120–250 nm). Varying the initial amount of PTX from 25 to 150 μg/mg of polymer ensured a 98–99% encapsulation efficiency and was accompanied by an increase in nanoparticle size at loadings over 50 μg/mg of polypeptide. The optimal polypeptide/siRNA ratio for Arg-containing polypeptides was 12, while for His-containing polypeptides it was 16. At these ratios, a total loading of siRNA was achieved. Co-loading PTX and nucleic acid resulted in the smallest compositions (90–200 nm) that were stable for at least 3 weeks at room temperature and in the presence of an extracellular physiological heparin concentration. The co-encapsulation of small double-stranded nucleic acid and PTX contributed to a decrease in the rate of cytostatic drug release. The formed empty polypeptide nanoparticles showed less cytotoxicity for normal cells (HEK 293T) and more for cancer cells (HeLa and A549). Both single-component formulations showed corresponding biological activity. All tested PTX formulations revealed the cell inhibitory effect (IC_50_ 4.5–6.2 ng/mL) comparable to free PTX (IC_50_ 5.1 ng/mL). At the same time, only one polypeptide system containing Tyr and Arg revealed the appropriate siRNA delivery (56–70% GFP knockdown) and gene silencing close to commercial transfecting agents (65–85% GFP knockdown). The advantages of the developed polypeptide systems are their total biodegradability to nontoxic metabolites and ability to joint-load drugs of different natures such as hydrophobic drugs and siRNA. However, among the considered polypeptides, P[KK(Y)K(R)]-based delivery systems can be considered the most promising for further in vivo codelivery of cytostatic and gene drugs due to the appropriate size of these delivery systems, their moderate cytotoxicity, and the in vitro biological efficacy of both encapsulated PTX and siRNA. Given that the loading of cytostatic drugs and siRNA is based on hydrophobic (in the case of PTX) and electrostatic (in the case of siRNA) interactions, any hydrophobic drug and siRNA can be successfully loaded into the developed systems.

## Figures and Tables

**Table 1:** Molecular-weight and hydrodynamic characteristics of synthesized poly-l-lysine and its derivatives obtained by modification with hydrophobic amino acids determined by SLS (DMF, 25 °C).

Polymer	*dn*/*dc ^a^*, cm^3^/g	*M_w_*	*A*_2_ *^b^*, cm^3^·mol/g^2^	*R_h-D_ ^c^*, nm
P[K(Z)]	0.1128	16,800	2.94 × 10^−3^	2.1
P[KK(V)]	0.1139	12,500	3.79 × 10^−3^	1.8
P[KK(I)]	0.1000	13,100	2.13 × 10^−2^	1.7
P[KK(Y)]	0.1342	14,000	5.34 × 10^−4^	1.2
P[KK(F)]	0.1151	n/c *^e^*	n/c *^e^*	0.8 *^d^*

*^a^* *dn*/*dc* is specific refractive index increment, i.e., the change in refractive index with concentration; *^b^ A*_2_ is the second virial coefficient in the Debye equation for static light scattering; *^c^ R_h-D_* is the hydrodynamic radius of the macromolecule calculated using the Einstein–Stocks equation; *^d^* Besides particles of this size, objects with a large hydrodynamic radius were also detected; *^e^* n/c means that the values were not calculated due to the presence of aggregates; P[K(Z)] is Z-protected poly(αl-lysine); P[KK(V)], P[KK(I)], P[KK(Y)], and P[KK(F)] are poly(αl-lysine) samples partly modified with l-valine, l-isoleucine, l-tyrosine, and l-phenylalanine, respectively.

**Table 2:** Composition of the amphiphilic copolymers obtained by the post-polymerization modification of poly-l-lysine with various hydrophobic and basic amino acids.

Polymer *^a^*	Hydrophobic Amino Acid Content (mol%)			Arg/His Content (mol%), HPLC		Calculated *M_n_^c^*
	Amino Acid	^1^H NMR	HPLC *^b^*	Arg	His	
P[KK(V)K(R)]	**Val**	21	26	39	-	19,890
P[KK(V)K(H)]				-	42	19,580
P[KK(I)K(R)]	**Ile**	25	25	32	-	19,390
P[KK(I)K(H)]				-	42	20,090
P[KK(Y)K(R)]	**Tyr**	16	16	35	-	19,620
P[KK(Y)K(H)]				-	43	20,020
P[KK(F)K(R)]	**Phe**	17	18	28	-	18,640
P[KK(F)K(H)]				-	43	20,050
P[KK(W)K(R)]	**Trp**	19	-	21	-	18,450
P[KK(W)K(H)]				-	41	20,630

*^a^* The amino-acid composition of polypeptides is given in the form of one-letter amino acid designations; *^b^* The averaged results of three independent hydrolyses of polymer samples followed by HPLC analysis; *^c^* The averaged values of hydrophobic amino acid content determined by both ^1^H NMR and HPLC were used for *M_n_* calculation.

**Table 3:** Correlation coefficients and dissolution model parameters obtained by mathematical modelling of PTX release from various PP@PTX systems.

Model	Correlation Coefficients and Parameters	P[KK(F)K(R)]@PTX		P[KK(I)K(R)]@PTX		P[KK(F)K(H)]@PTX	
		7 h	240 h	7 h	240 h	7 h	240 h
**Zero-order** *F = k*_0_ * t	*R* ^2^	0.9955	0.7281	0.9968	0.7945	0.9858	0.7826
	*k*_0_	1.085	0.058	1.232	0.069	0.845	0.068
**First-order** *F =* 100 * [1 − *Exp*(*−k*_1_ * t)]	*R* ^2^	0.9960	0.7357	0.9974	0.8046	0.9872	0.7940
	*k*_1_	7.5 × 10^−3^	6.3 × 10^−4^	9.7 × 10^−3^	9.0 × 10^−4^	8.7 × 10^−3^	7.5 × 10^−4^
**Higuchi** F = k_H_ * t ^0.5^	*R* ^2^	0.9957	0.8466	0.9915	0.8975	0.9991	0.9095
	*k_H_*	2.345	0.871	2.625	1.184	1.883	0.983
**Korsmeyer–Peppas** F = k_KP_ * t^n^	*R* ^2^	0.9966	0.9249	0.9963	0.9497	0.9992	0.9583
	*k_KP_*	2.227	3.504	2.158	3.807	1.757	2.826
	*n*	0.538	0.207	0.643	0.255	0.551	0.277
**Hixon–Crowell** *F* = 100 * [1 − (1 − k_HC_ * t)^3^]	*R* ^2^	0.9960	0.7332	0.9973	0.8012	0.9867	0.7902
	*k_HC_*	3.7 × 10^−3^	2.0 × 10^−4^	4.2 × 10^−3^	2.9 × 10^−4^	2.8 × 10^−3^	2.4 × 10^−4^
**Hopfenberg** *F* = 100 * [1 − (1 − k_HB_ * t)*^n^*]	*R* ^2^	0.9963	0.7357	0.9975	0.8045	0.9871	0.7939
	*k_HB_*	8.7 × 10^−5^	5.1 × 10^−6^	4.2 × 10^−4^	3.9 × 10^−6^	4.7 × 10^−4^	6.1 × 10^−6^
**Baker–Lonsdale** 3/2 * [1 − (1 − *F*/100)^(2/3)^] − *F*/100 = *k_BL_* * *t*	*R* ^2^	0.9953	0.8497	0.9911	0.9011	0.9990	0.9129
	*k_BL_*	9.4 × 10^−5^	1.3 × 10^−5^	11.2 × 10^−4^	3.5 × 10^−5^	6.0 × 10^−5^	1.7 × 10^−5^
**Weibull** *F* = 100 * {1 − *Exp*[−((*t − Ti*)*^β^*)/*α*]}	*R* ^2^	0.9990	0.9432	0.9987	0.9616	0.9992	0.9683
	α	61.154	25.591	23.529	5.71	56.679	31.695
	β	0.694	0.195	0.248	0.24	0.562	0.267
**Gompertz** *F* = 100 * *Exp*{−α * Exp[−*β* * *log*(*t*)]}	*R* ^2^	0.9910	0.9406	0.9904	0.9656	0.9979	0.9737
	α	3.815	3.423	3.869	3.392	4.063	3.716
	β	0.374	0.181	0.465	0.247	0.366	0.248
**Peppas–Sahlin** *F = k*_1_ * *t^m^ + k*_2_ * *t*^(2*m)^	*R* ^2^	0.9979	0.9785	0.9974	0.9815	0.9993	0.9925
	*k*_1_	1.654	3.115	1.546	3.290	1.789	2.256
	*k*_2_	0.590	0.240	0.648	0.194	0.041	0.110
	*m*	0.400	0.397	0.458	0.431	0.578	0.466

**Table 4:** Values of IC_50_ determined for various polypeptide particles incubated with normal (HEK 293) and cancer (HeLa, A549) cells (MTT, 72 h).

Polypeptide Particles	IC_50_ (µg/mL)		
	HEK 293T	HeLa	A549
P[KK(V)K(R)]	-	71 ± 14	25 ± 3
P[KK(V)K(H)]	254 ± 44	92 ± 23	48 ± 6
P[KK(I)K(R)]	-	57 ± 3	28 ± 4
P[KK(I)K(H)]	233 ± 48	83 ± 13	93 ± 9
P[KK(Y)K(R)]	134 ± 28	55 ± 12	47 ± 8
P[KK(Y)K(H)]	183 ± 16	55 ± 7	57 ± 13
P[KK(F)K(R)]	-	88 ± 13	58 ± 9
P[KK(F)K(H)]	234 ± 38	118 ± 22	90 ± 18
P[KK(W)K(R)]	289 ± 88	-	69 ± 8
P[KK(W)K(H)]	306 ± 95	-	134 ± 19

**Table 5:** Values of IC_50_ determined for various PTX-loaded polypeptide particles incubated with cancer cells (A549 cell line, MTT, 72 h).

System	IC_50_ (ng/mL)
	A549
Free PTX	5.1 ± 1.9
P[KK(I)K(R)]@PTX	6.2 ± 2.3 *
P[KK(I)K(H)]@PTX	4.7 ± 0.5 *
P[KK(Y)K(R)]@PTX	4.9 ± 1.5 *
P[KK(Y)K(H)]@PTX	4.4 ± 0.6 *
P[KK(F)K(H)]@PTX	5.6 ± 1.2 *
P[KK(W)K(H)]@PTX	4.5 ± 1.6 *

* the difference with control (free PTX) were non-significant (*p* ≥ 0.05).

## Data Availability

The data supporting the findings of this study are available within the article or its Supplementary Materials.

## Supplementary Materials

The following supporting information can be downloaded at: https://www.mdpi.com/article/10.3390/pharmaceutics15041308/s1, Figure S1: ^1^H NMR spectra of P[K(Z)] (**A**) and P[K] (**B**) (DMSO-d6 for (**A**) and D_2_O for (**B**), 25 °C); Figure S2: ^1^H NMR spectra of P[KK(F)] (**A**), P[KK(V)] (**B**), P[KK(I)] (**C**), P[KK(Y)] (**D**) and P[KK(W)] (**E**) (DMSO-d6, 25 °C); Figure S3: ^1^H NMR spectra of P[KK(Y)K(H)] (**A**), P[KK(Y)K(R)] (**B**), P[KK(I)K(H)] (**C**), P[KK(I)K(R)] (**D**) (DMSO-d6, 25 °C); Figure S4: Best model fittings for PTX release from various polypeptide formulations under study; Table S1: Lipophilicity of certain amino acids calculated from experimental results elsewhere; Figure S5: Cell viability assay of various polypeptide particles using normal and cancer cells (MTT, 72 h): (**A**)—HEK 293T, (**B**)—HeLa, and (**C**)—A549 cell lines; Figure S6: Dose–response curves for free PTX (**A**) and certain of its polypeptide formulations (**B**–**D**): P[KK(I)K(H)]@PTX (**B**), P[KK(Y)K(H)]@PTX (**C**), and P[KK(W)K(H)]@PTX (**D**).
