# Supplementary material for: Amphiphilic Polypeptides Obtained by Post-Polymerization Modification of Poly-l-Lysine as Systems for Combined Delivery of Paclitaxel and siRNA
Source: Pharmaceutics. 2023 Apr 21;15(4):1308. doi: 10.3390/pharmaceutics15041308 (PMC10143851; doi:10.3390/pharmaceutics15041308)
Supplement: Supplementary file 1 [file pharmaceutics-15-01308-s001.zip › pharmaceutics-2296866-supplementary.pdf]

pharmaceutics

Supplementary Materials

Amphiphilic Polypeptides Obtained by Post-Polymerization Modification of Poly-L-Lysine as Systems for Combined Delivery of Paclitaxel and siRNA

Apollinariia Dzhuzha [1,2], Erik Gandalipov [3], Viktor Korzhikov-Vlakh [1], Elena Katernyuk [1,2], Natalia Zakharova [2], Sergey Silonov [1,4], Tatiana Tennikova [1] and Evgenia Korzhikova-Vlakh [1,2,*]

[1] Institute of Chemistry, Saint-Petersburg State University, Universitetsky Pr. 26, St. Petersburg 198504, Russia; polinadzhuzha@mail.ru (A.D.); v_korzhikov@mail.ru (V.K.-V.); st068478@student.spbu.ru (E.K.); silonovsa25@yandex.ru (S.S.); tennikova@mail.ru (T.T.)
[2] Institute of Macromolecular Compounds, Russian Academy of Sciences, Bolshoy Pr. 31, St. Petersburg 199004, Russia; zakharova@gmail.com
[3] International Institute of Solution Chemistry and Advanced Materials Technologies, ITMO University, Lomonosov Street 9, St. Petersburg 191002, Russia; gandalipov@scamt-itmo.ru
[4] Institute of Cytology, Russian Academy of Sciences, Tihkorezky Pr. 4, St. Petersburg 194064, Russia
* Correspondence: vlakh@hq.macro.ru

* Correspondence: vlakh@hq.macro.ru

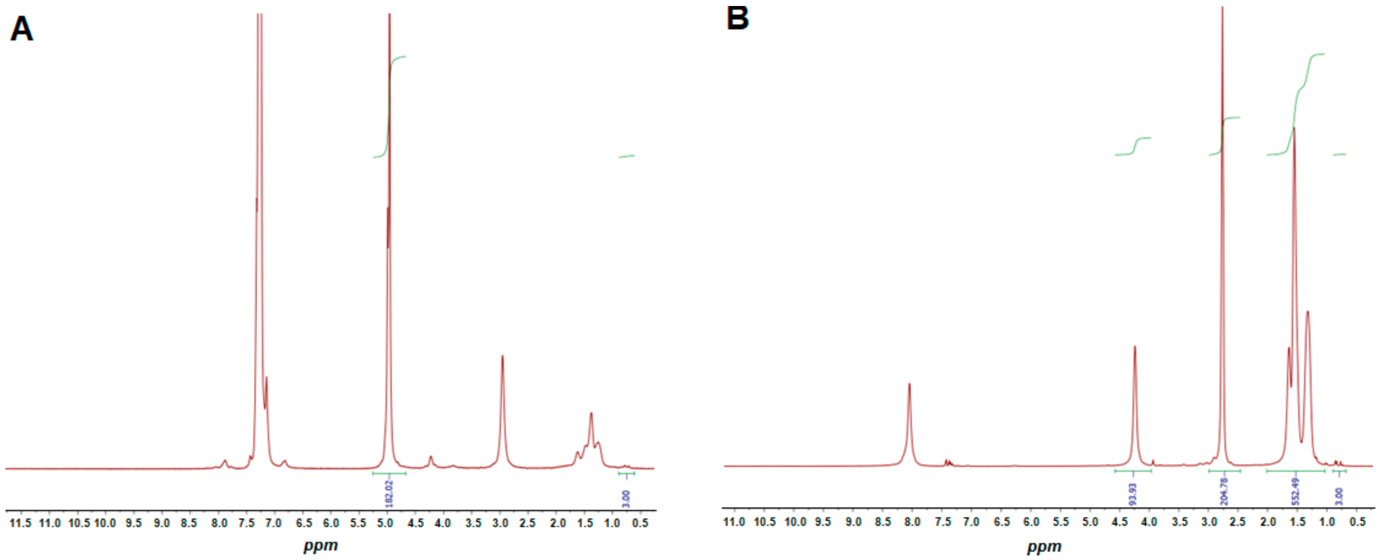

Figure S1. ^{1}H NMR spectra of P[K(Z)] (A) and P[K] (B) (DMSO-d6 for (A) and D_2O for (B), 25 °C). In spectra: *(δ, ppm)*: 0.83 (C<u>H</u>$_3$, hexylamine), 1.0-2.0 (3C<u>H</u>$_2$, Lys), 2.5-3.0 (C<u>H</u>$_2$, Lys), 4.0-4.4 (C<u>H</u>, Lys); 4.7-5.2 (-C<u>H</u>$_2$-C_6H_5, Z-group) (A). The degree of polymerization (DP) is 91. Z-group signals are completely absent from the B spectrum, indicating complete deprotection.

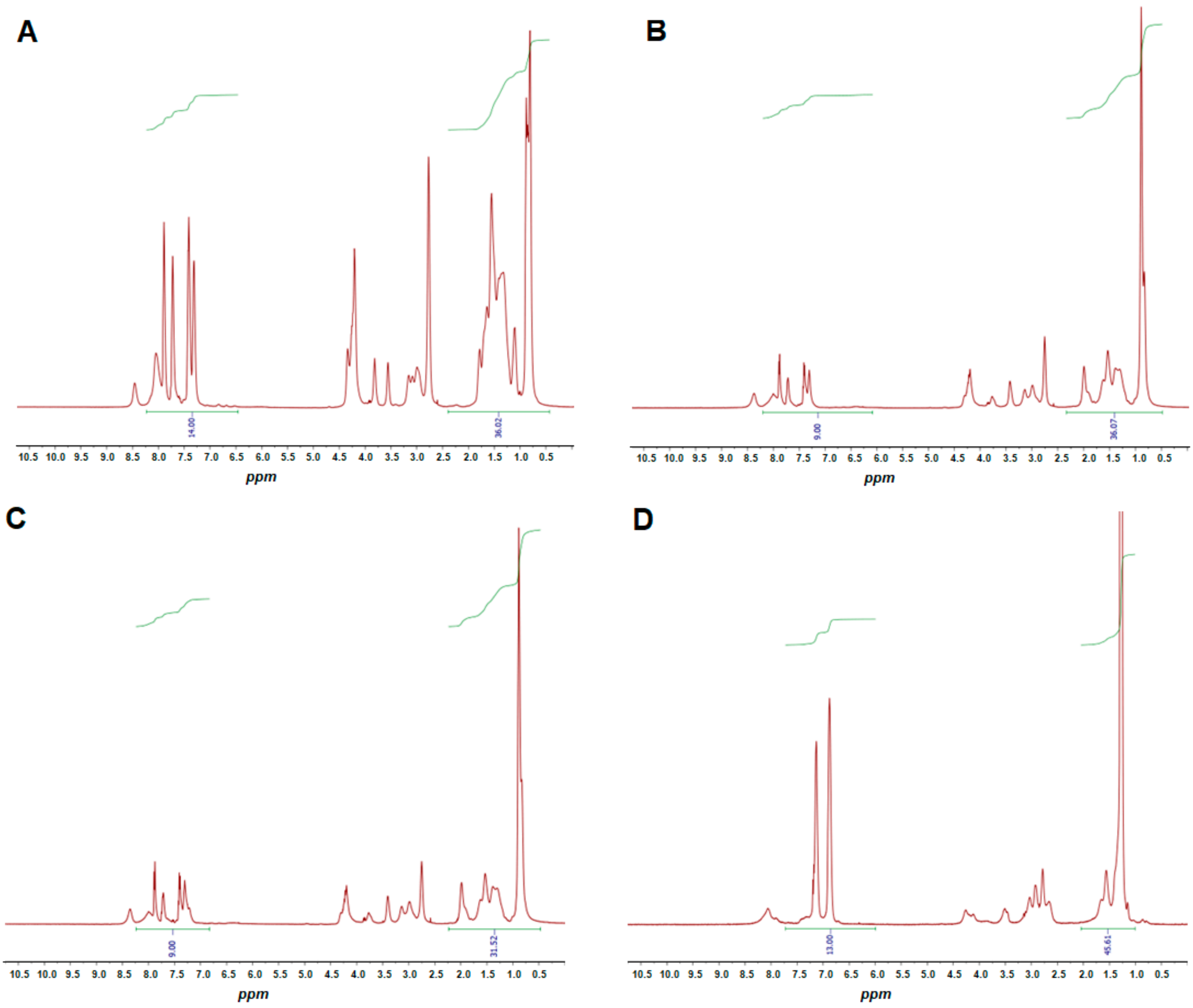

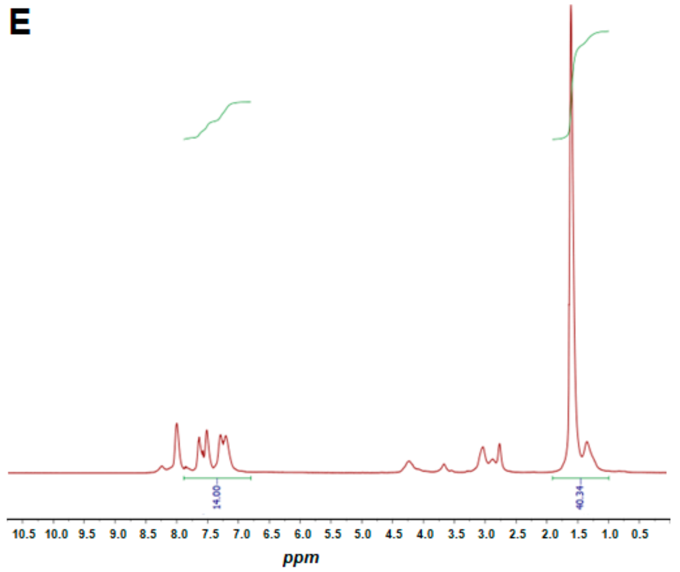

Figure S2. ^{1}H NMR spectra of P[KK(**F**)] **(A)**, P[KK(**V**)] **(B)**, P[KK(**I**)] **(C)**, P[KK(**Y**)] **(D)** and P[KK(**W**)] **(E)** (DMSO-d6, 25 °C).

Signals corresponding to main chain and Fmoc-protected hydrophobic amino acids *(δ, ppm)*: **(A)** 0.5–2.4 (3CH_2, Lys); 6.4–8.2 (8H, Fmoc; 1H, NH; C_6H_5, Phe); **(B)** 0.5–2.4 (3CH_2, Lys; 2CH_3 + CH, Val); 6.4–8.2 (8H, Fmoc; 1H, NH); **(C)** 0.5–2.0 (3CH_2, Lys; 2CH_3 + CH_2, Ile); 6.4–8.2 (8H, Fmoc; 1H, NH); **(D)** 0.5–2.0 (3CH_2, Lys; 3CH_3, tBu); 6.4–7.5 (8H, Fmoc; 1H, NH; C_6H_4, Tyr); **(E)** 0.5–2.0 (3CH_2, Lys; 3CH_3, BOC); 6.4–8.2 (8H, Fmoc; 1H, NH; C_8H_5, Trp.

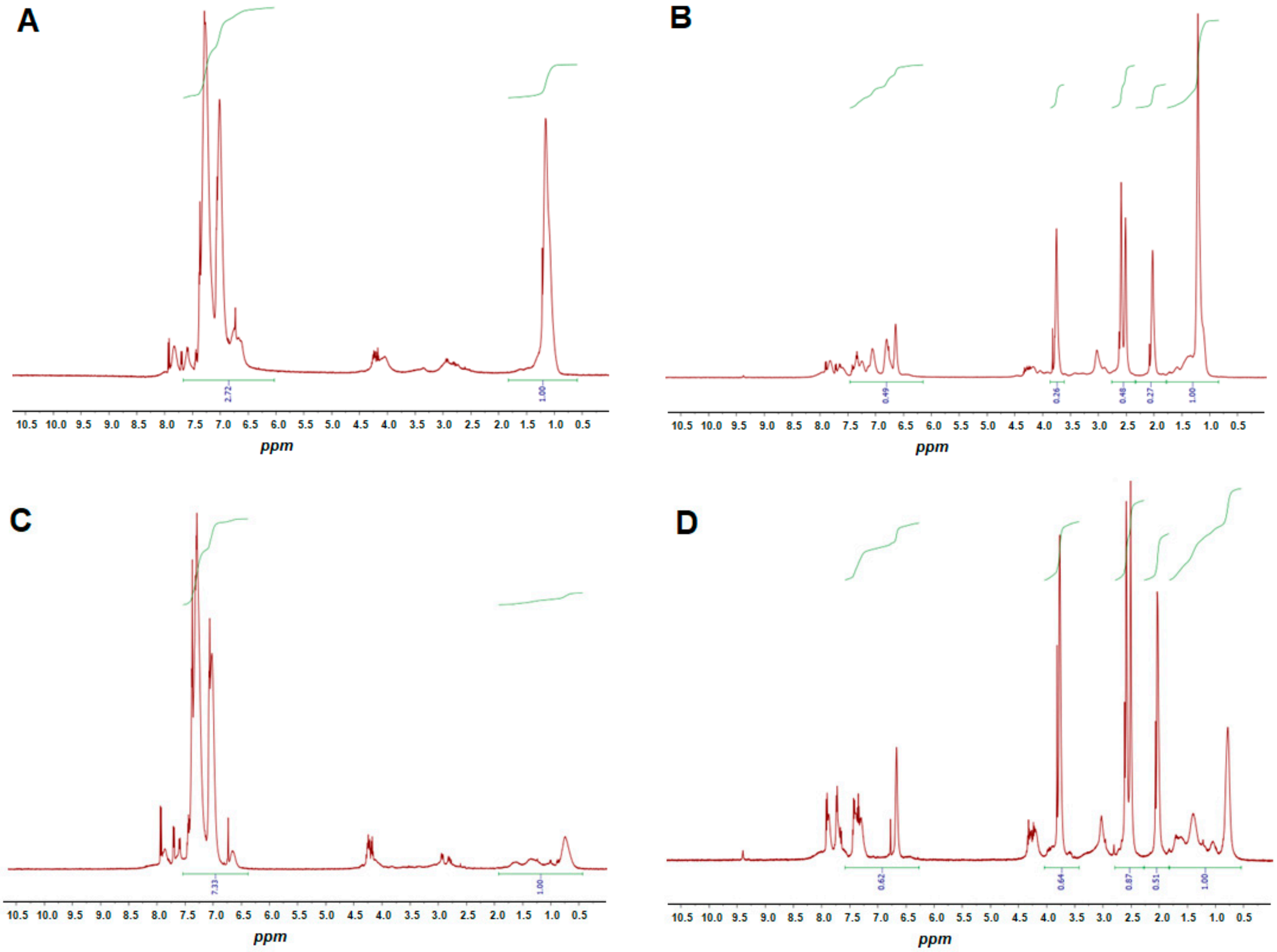

Figure S3. ^{1}H NMR spectra of P[KK(**Y**)K(**H**)] **(A)**, P[KK(**Y**)K(**R**)] **(B)**, P[KK(**I**)K(**H**)] **(C)**, P[KK(**I**)K(**R**)] **(D)** (DMSO-d6, 25 °C). Signals corresponding to main chain and protected amino acids in the side chain *(δ, ppm)*: **(A)** 0.5–2.0 (3CH_2, Lys; 3CH_3, tBu); 6.4–7.5 (8H, Fmoc; 1H, NH; C_6H_4, Tyr; 3C_6H_5, Trt; 2H, His); **(B)** 0.5–1.8 (3CH_2, Lys; 3CH_3, tBu; 2CH_2, Arg); 1.8-2.3 (CH2, Arg); 2.4-2.7 (3CH_3, Mtr); 3.5-3.9 (CH_3-O, Mtr); 6.4-7.5 (8H, Fmoc; 1H, NH; C_6H_4, Tyr; H-Ar, Arg); **(C)** 0.5–2.0 (3CH_2, Lys; 2CH_3 + CH_2, Ile); 6.4–8.2 (8H, Fmoc; 1H, NH; 3C_6H_5, Trt; 2H, His); **(D)** 0.5–1.8 (3CH_2, Lys; 2CH_3 + CH_2, Ile; 2CH_2, Arg); 1.8-2.3 (CH2, Arg); 2.4-2.7 (3CH_3, Mtr); 3.5-4.0 (CH_3-O, Mtr); 6.4-8.2 (8H, Fmoc; 1H, NH; H-Ar, Arg).

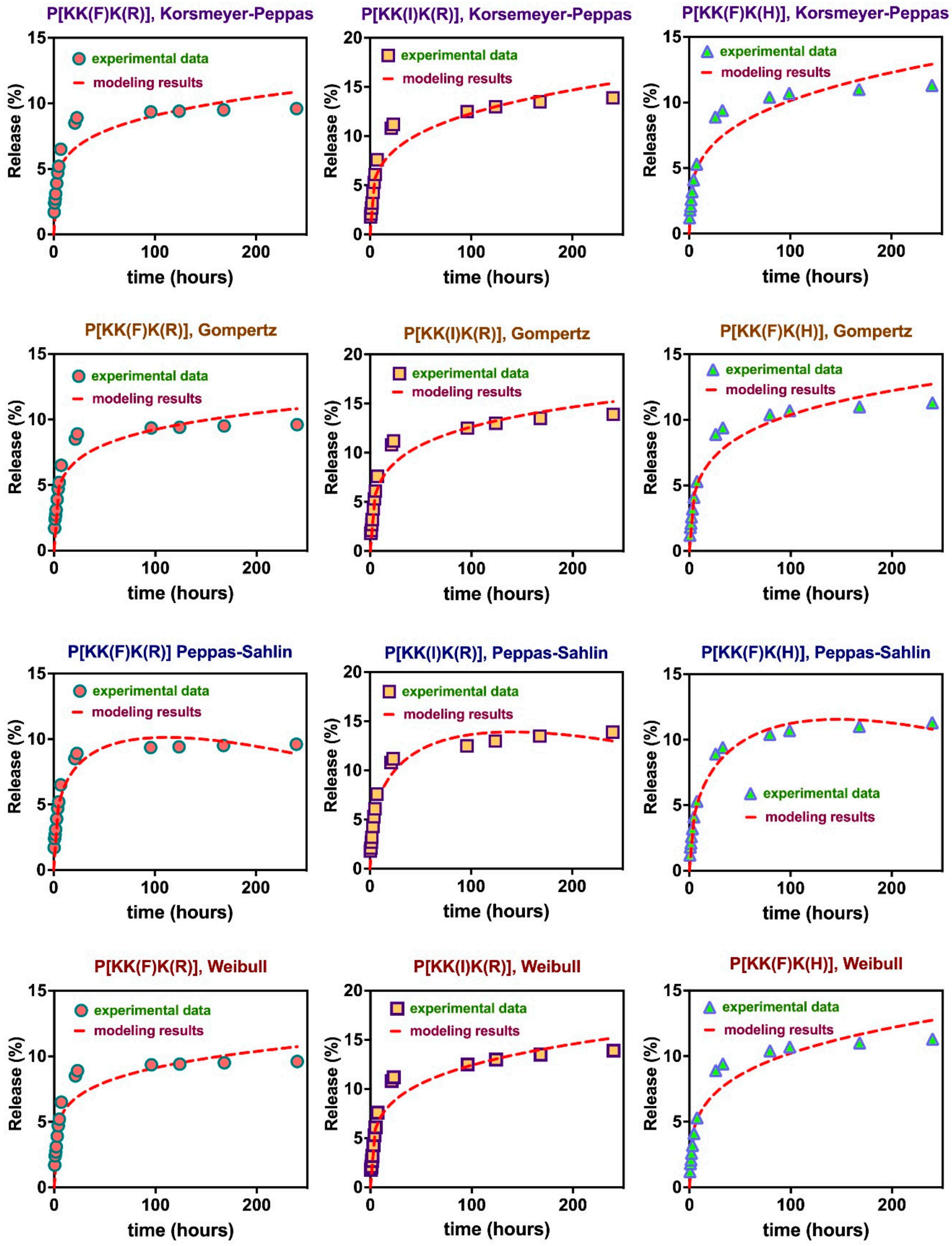

Figure S4. Best model fittings for PTX release from various polypeptide formulations under study.

Table S1. Lipophilicity of some amino acids calculated from experimental results elsewhere.

Amino acid	Lipophilicity	
	**Parker et al.** [1]	**Chmelik et al.** [2]
Tyrosine (Tyr, Y)	1.9	0.85
Valine (Val, V)	3.7	1.07
Isoleucine (Ile, I)	8.0	1.53
Phenylalanine (Phe, F)	9.2	1.91
Tryptophan (Trp, W)	10.0	2.01

References

1. Parker, J.M.R.; Guo, D.; Hodges, R.S. New hydrophilicity scale derived from high-performance liquid chromatography peptide retention data: correlation of predicted surface residues with antigenicity and x-ray-derived accessible sites. *Biochemistry* **1986**, *25*, 5425–5432.
2. Chmelík, J.; Hudeček, J.; Putyera, K.; Makovička, J.; Kalous, V.; Chmelíková, J. Characterization of the hydrophobic properties of amino acids on the basis of their partition and distribution coefficients in the 1-octanol-water system. *Collect. Czechoslov. Chem. Commun.* **1991**, *56*, 2030–2041.

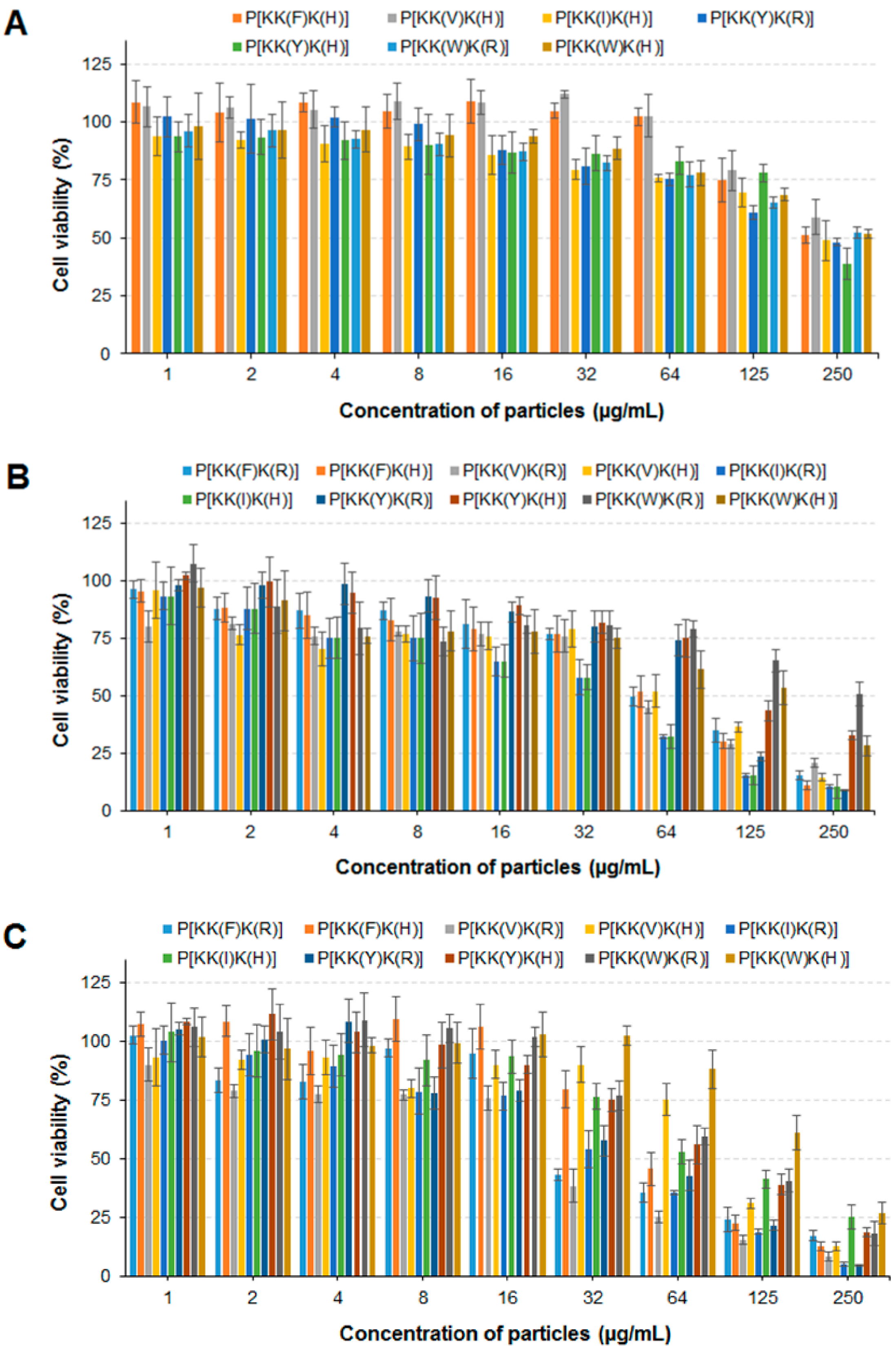

Figure S5. Cell viability assay of various polypeptide particles using normal and cancer cells (MTT, 72 h): A – HEK 293T, B – HeLa and C – A549 cell lines.

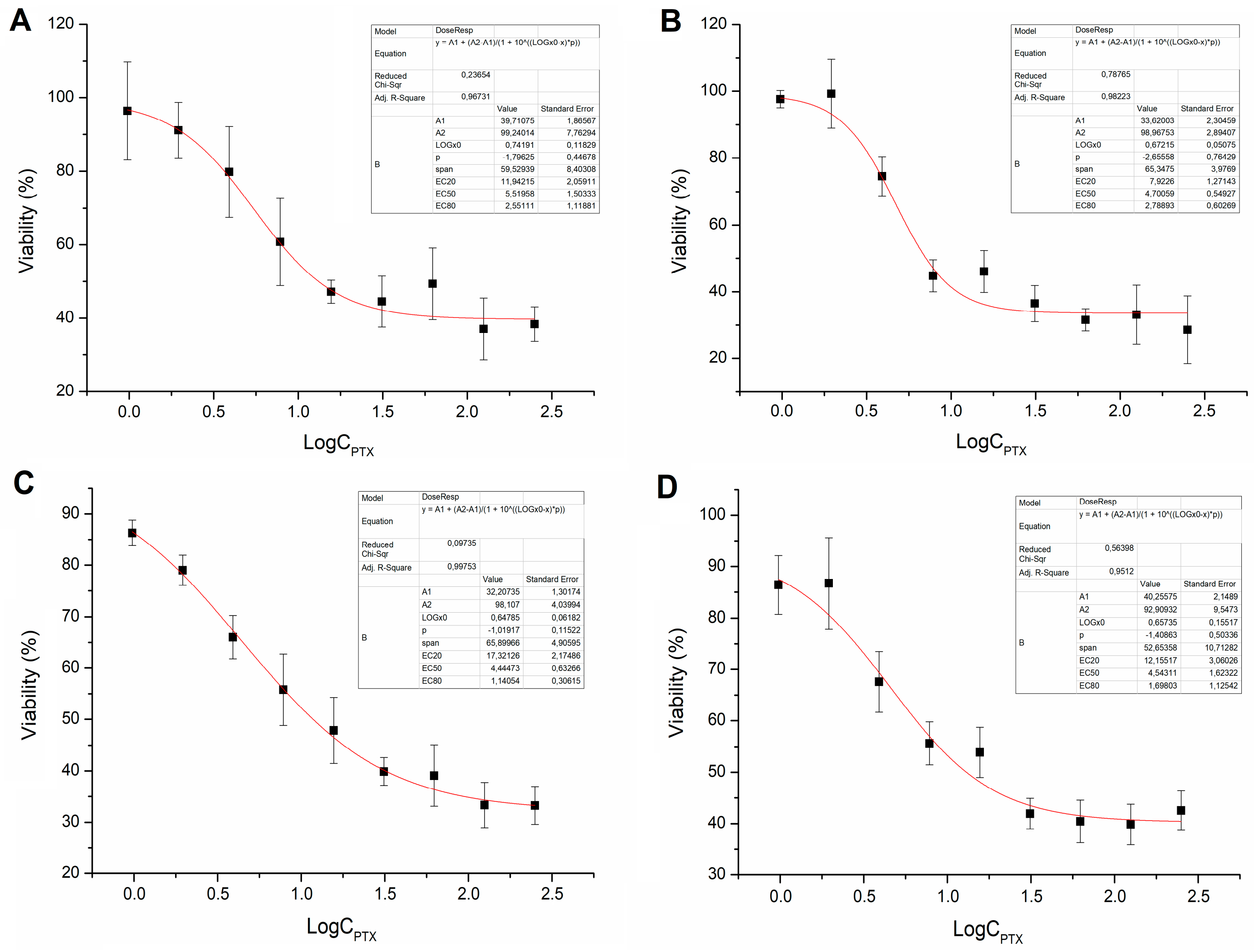

Figure S6. Dose-response curves for free PTX **(A)** and some its polypeptide formulations **(B-D)**: P[KK(**I**)K(**H**)]@PTX **(B)**, P[KK(**Y**)K(**H**)]@PTX **(C)** and P[KK(**W**)K(**H**)]@PTX **(D)**.
